# The mecillinam resistome reveals a role for peptidoglycan endopeptidases in stimulating cell wall synthesis in *Escherichia coli*

**DOI:** 10.1371/journal.pgen.1006934

**Published:** 2017-07-27

**Authors:** Ghee Chuan Lai, Hongbaek Cho, Thomas G. Bernhardt

**Affiliations:** Department of Microbiology and Immunobiology, Harvard Medical School, Boston, MA, United States of America; Uppsala University, SWEDEN

## Abstract

Bacterial cells are typically surrounded by an net-like macromolecule called the cell wall constructed from the heteropolymer peptidoglycan (PG). Biogenesis of this matrix is the target of penicillin and related beta-lactams. These drugs inhibit the transpeptidase activity of PG synthases called penicillin-binding proteins (PBPs), preventing the crosslinking of nascent wall material into the existing network. The beta-lactam mecillinam specifically targets the PBP2 enzyme in the cell elongation machinery of *Escherichia coli*. Low-throughput selections for mecillinam resistance have historically been useful in defining mechanisms involved in cell wall biogenesis and the killing activity of beta-lactam antibiotics. Here, we used transposon-sequencing (Tn-Seq) as a high-throughput method to identify nearly all mecillinam resistance loci in the *E*. *coli* genome, providing a comprehensive resource for uncovering new mechanisms underlying PG assembly and drug resistance. Induction of the stringent response or the Rcs envelope stress response has been previously implicated in mecillinam resistance. We therefore also performed the Tn-Seq analysis in mutants defective for these responses in addition to wild-type cells. Thus, the utility of the dataset was greatly enhanced by determining the stress response dependence of each resistance locus in the resistome. Reasoning that stress response-independent resistance loci are those most likely to identify direct modulators of cell wall biogenesis, we focused our downstream analysis on this subset of the resistome. Characterization of one of these alleles led to the surprising discovery that the overproduction of endopeptidase enzymes that cleave crosslinks in the cell wall promotes mecillinam resistance by stimulating PG synthesis by a subset of PBPs. Our analysis of this activation mechanism suggests that, contrary to the prevailing view in the field, PG synthases and PG cleaving enzymes need not function in multi-enzyme complexes to expand the cell wall matrix.

## Author summary

Penicillin and related beta-lactams are one of our oldest and most effective classes of antibiotics. These drugs target enzymes called penicillin-binding proteins (PBPs) that build the essential cell wall that surrounds bacterial cells. Beta-lactams have long been used as chemical and genetic probes to uncover the mechanisms required for proper bacterial cell wall biogenesis. In this report, we use a high-throughput genetic approach to comprehensively identify nearly all genetic loci that promote resistance to the beta-lactam mecillinam in the model organism *Escherichia coli*. Moreover, by performing our analysis in several different genetic backgrounds we were able to generate a rich resource that defines those alleles that promote resistance by inducing a stress response and those that are more likely to do so by directly modulating cell wall synthesis. Further characterization of one of the stress response-independent resistance loci helped us discover that enzymes that cleave crosslinks in the cell wall are capable of activating cell wall synthesis by a subset of PBPs. Our analysis of the activation mechanism challenges the prevailing view in the field that cell wall synthases and cell wall cleaving enzymes must work in multi-enzyme complexes to assemble the cell wall.

## Introduction

Bacterial cells are typically surrounded by an essential net-like macromolecule called the cell wall. This structure is constructed of peptidoglycan (PG), a unique bacterial heteropolymer consisting of glycan chains of N-acetylmuramic acid (MurNAc) and N-acetylglucosamine (GlcNAc) repeating units with attached stem-peptides used to form the matrix crosslinks [1]. Many of our most effective antibiotic therapies target cell wall biogenesis, and much of what we know about the cell wall assembly process was facilitated using these antibiotics as functional probes. In both respects, penicillin and related beta-lactam drugs are standouts. They are the most frequently prescribed antibiotics worldwide, and their use in basic research has provided major insights into the structure of the wall and how it is built.

Beta-lactams inhibit their targets by covalently modifying their active sites [2], a property that facilitated the identification of the penicillin-binding proteins (PBPs) as key cell wall biogenesis factors. The PBPs are subdivided into class A (aPBPs), class B (bPBPs), and class C (cPBPs) enzymes [3]. aPBPs are bifunctional and possess both glycosyltransferase (GT) activity for polymerizing the glycan strands of PG and transpeptidase (TP) activity for crosslinking them. bPBPs, on the other hand, are only known to possess TP activity [3]. cPBPs typically cleave PG and either break crosslinks (endopeptidases) or tailor the peptide stem by removing the terminal D-Ala residue (carboxypeptidases) [3]. Beta-lactams block the TP active site of the synthetic PBPs and inhibit PG hydrolysis by the cPBPs. Depending on the type and concentration of beta-lactam used, cells treated with these drugs either rapidly lyse or undergo significant morphological changes before lysing several generations after drug addition[4]. Despite years of study, molecular details of the events downstream of PBP inhibition that elicit these dramatic effects are only beginning to be uncovered.

Beta-lactams that are highly specific for a single target PBP have been particularly useful probes for understanding PG biogenesis and the beta-lactam killing mechanism. Among them, mecillinam has probably stimulated the greatest number of discoveries. It specifically targets the bPBP PBP2 in *Escherichia coli* and causes the loss of rod shape and the formation of large spherical cells that eventually lyse [4]. Early selections for mecillinam resistance in *E*. *coli* led to the identification of loss-of-function mutations in the *mrdAB* genes (a.k.a. *pbpA* and *rodA*, encoding PBP2 and RodA) and *mreCDE* genes [4–6]. These mutants paved the way for the discovery of the cell wall biogenesis machinery called the Rod system (elongasome) [1]. This system promotes the elongation of rod-shaped cells and is organized by dynamic filaments of the actin homolog MreB. Within the complex, the SEDS (shape, elongation, division, and sporulation)-family [7] protein RodA supplies the PG polymerase function while PBP2 uses its TP activity to crosslink the new material into the PG matrix [8,9]. An analogous multi-protein machine called the divisome mediates PG synthesis during cell division [1]. It is organized by the tubulin-like FtsZ protein, which brings together a subset of PG biogenesis factors similar to those in the Rod system, including the SEDS-family protein FtsW and PBP3, a bPBP related to PBP2.

The Rod system is normally essential in *E*. *coli* [10,11]. However, when this essentiality was initially discovered, it conflicted with the original reports describing the isolation of mecillinam-resistant mutants defective for cell shape and Rod system activity [4,5]. It was subsequently shown that these mutant isolates had secondary mutations that increased the production of FtsZ to suppress Rod system essentiality [6,10,11]. The reason why extra FtsZ (designated FtsZ^up^) results in suppression is not clear. Nevertheless, the phenomenon suggested that the original selections for mecillinam resistance were more complicated than initially appreciated. If mecillinam works simply by inactivating the Rod system, why isn't FtsZ overproduction alone sufficient to bypass drug action and promote resistance? Why were double mutants that both overproduce FtsZ and inactivate the Rod system isolated?

This genetic conundrum led us to reinvestigate the mode-of-action of mecillinam. We discovered that mecillinam not only inhibits the TP activity of PBP2 but also causes the activity of the Rod system to become toxic [12]. Thus, to gain mecillinam resistance, cells must both inactivate the Rod system and acquire mutations that render the system non-essential for growth. The toxic activity of the Rod system in the presence of mecillinam is caused by the inactivation of PBP2 and the failure to crosslink nascent PG material into the wall. The uncrosslinked glycans produced by the machine are rapidly degraded by the lytic transglycosylase (LT) Slt, resulting in a futile cycle of PG synthesis and degradation by the drug-targeted Rod complex [12]. Experiments with the beta-lactams cephalexin and cefsulodin showed that they also promote nascent PG degradation by the PG synthase systems they target, indicating that futile cycle induction is a common activity of this drug class in *E*. *coli* and likely many other gram-negative bacteria [12].

The downstream steps via which the futile cycle of PG synthesis and degradation induced by beta-lactams results in cell death and lysis have not been clearly defined. We reasoned that mutants resistant to the toxic effects of mecillinam should shed light on this lethal mode-of-action. Such mutants should also provide new insights into drug resistance mechanisms and the process of cell wall biogenesis in general. Many mecillinam-resistant *E*. *coli* mutants have been isolated and characterized previously, including several from the extensive studies of D’Ari and co-workers [4–6,13–18]. However, these mutants were selected under conditions where the Rod system was essential. Thus, they were required to survive both the crippling of the Rod system by PBP2 inactivation and the downstream toxic effects of the futile cycle. These conditions likely limited the spectrum of mutants isolated.

To overcome the complications of prior genetic analyses, we initiated selections for mecillinam resistance using FtsZ^up^ cells, in which the Rod system is non-essential. Thus, in order to grow, mutants are only required to survive the futile cycle of PG synthesis and degradation. Under these conditions, mecillinam-resistant mutants arise at a high frequency, indicating that there are many ways to either inactivate the futile cycle or ameliorate the problems it causes. Therefore, to identify the full spectrum of resistance loci, we employed transposon sequencing (Tn-Seq) [19] of large pools of mutants capable of growth on either low, intermediate, or high doses of drug. Furthermore, because induction of the stringent response or envelope stress responses are known to provide protection from mecillinam lethality [6,20], we additionally performed the analysis of mecillinam resistance in genetic backgrounds defective for these responses. This approach allowed us to identify loci that most likely provide resistance via induction of the stress response systems. The results thus provide a useful dataset of mutants that are likely to be constitutively activated for the Rcs and stringent responses.

For further biological studies, we were especially interested in mecillinam-resistant mutants that appeared to be stress response-independent. We suspected that such loci are more likely to identify factors directly involved in modulating cell wall biogenesis to affect drug sensitivity. Among this class of mutants were those inactivated for the periplasmic protease Prc, which was recently implicated in the degradation of the cell wall endopeptidase MepS (Spr) [21,22]. We therefore hypothesized that elevated MepS concentration might provide mecillinam resistance. Strikingly, we found that overproduction of MepS and several other endopeptidases confers mecillinam resistance. This was a surprising result because PG degrading enzymes are typically associated with the induction of cell lysis following beta-lactam treatment, not with promoting survival [23]. Radiolabeling experiments showed that *mepS* overexpression stimulates PG synthesis by the aPBP PBP1b, which probably redirects PG precursor flux away from the Rod system to limit the futile cycle and promote mecillinam resistance. Our analysis of this activation mechanism suggests that, contrary to the prevailing view in the field [1,24,25], PG synthases and PG cleaving enzymes need not function in multi-enzyme complexes to expand the cell wall matrix.

## Results

### Identification of mecillinam resistance loci using Tn-Seq

**Fig 1:**
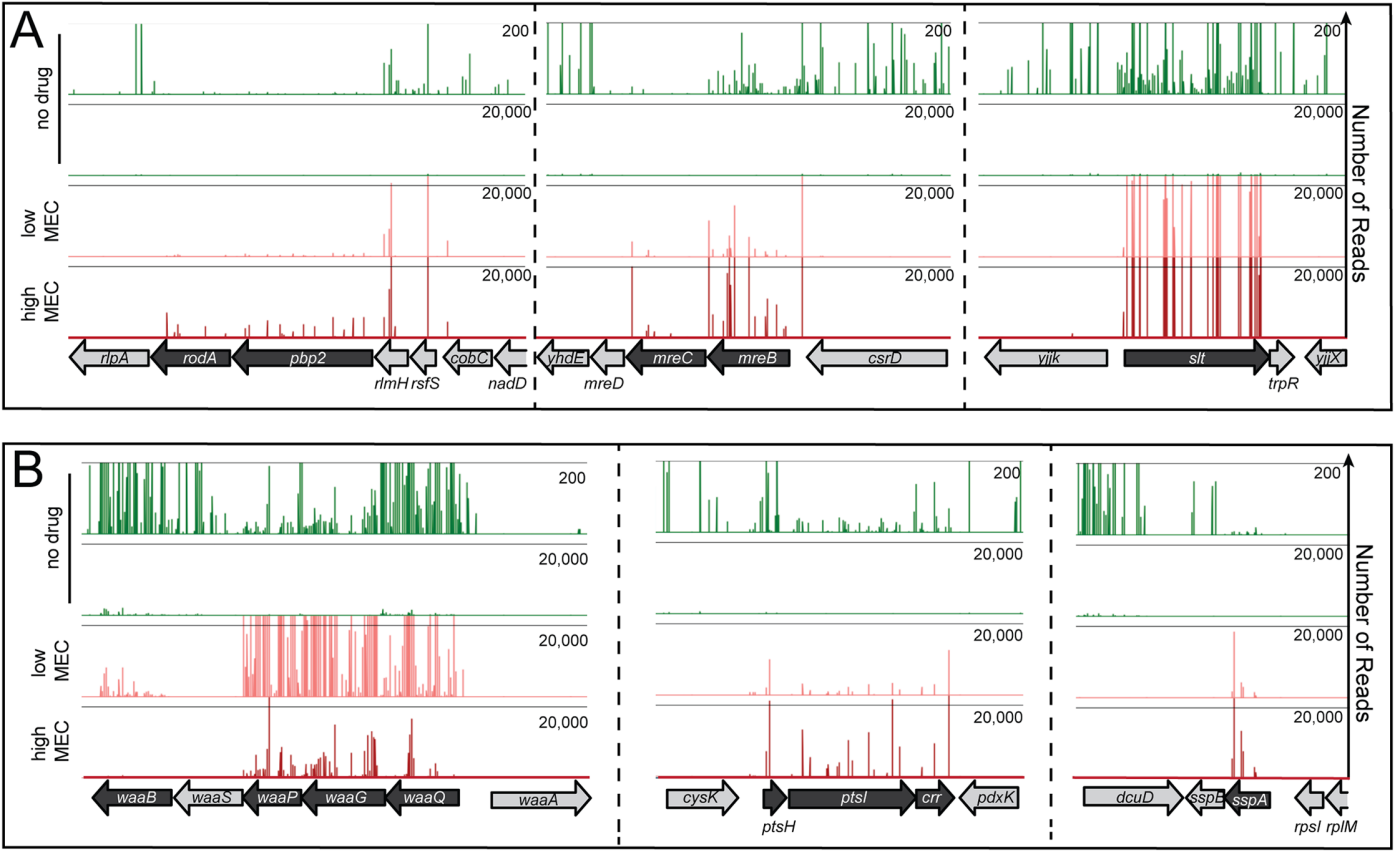
Tn-Seq profiles for mecillinam resistance loci. Shown are profiles of transposon insertions mapped by Tn-Seq for MG1655/pTB63 [WT/ftsZ^up^] transposon libraries grown without treatment (no drug) or harvested following growth on agar containing 1 or 10 μg/ml mecillinam (low and high MEC, respectively). In the profiles, the reading frames are indicated at the bottom of the panel. Transposon insertion sites are indicated by lines above the reading frames with their height reflecting the number of reads for each insertion. A. Profiles for the known mecillinam-resistance loci: mrdAB (pbp2 rodA), mreBCD, and slt. Note that mutants defective in mrdAB (pbp2 rodA), or mreBCD have a slow growth phenotype even in FtsZ^up^ cells such that insertions in these genes are underrepresented in the original library B. Profiles for novel mecillinam-resistance loci: waaQ-P, ptsHI, and sspA. Note that the profiles from no drug samples are displayed with a 200 read maximum (upper) and a 20,000 read maximum (lower) to show both the overall transposon insertion distribution in the original library and the magnitude of enrichment observed in the drug treated samples. A complete list of resistance loci and their enrichment ratios (comparing total reads for all insertions with and without drug) is given in S1 Table.

For our analysis, we used wild-type *E*. *coli* MG1655 cells producing extra FtsZ from a low-copy number plasmid containing the *ftsQAZ* operon (pTB63) [12]. When these FtsZ^up^ cells were selected for spontaneous mecillinam resistance at concentrations between 1–10 μg/ml, survivors arose at a frequency of 1–5 x 10^−4^. Similar selections using cells harboring a control plasmid (pSC101) yielded resistant mutants at a frequency of 0.8–1 x 10^−5^. The increased frequency of survival conferred by pTB63 indicated that previous selections for mecillinam resistance without elevated FtsZ levels were likely to have missed a significant number of resistance loci. To identify the full set of mecillinam resistance determinants, MG1655/pTB63 cells were mutagenized with the EZTn-Kan transposome to generate a library consisting of approximately 2 x 10^5^ independent insertions. The library was then plated on agar with 0, 1.0, 2.5, or 10 μg/ml mecillinam. Survivors on mecillinam arose at a frequency of 2–6 x 10^−3^. This frequency was ten times greater than for unmutagenized cells, indicating that the vast majority of the isolates gained resistance due to a transposon insertion. Given the high frequency of resistance, we expected the number of loci involved to be large. Therefore, rather than mapping individual alleles in isolated clones, we pooled the survivors at each mecillinam concentration and simultaneously mapped the location of all transposon insertions in the population using Tn-Seq methods [26]. Genes with an elevated frequency of transposon insertions in the mecillinam-treated samples relative to the untreated library were identified as likely resistance loci. Those identified as resistance loci on 10 μg/ml mecillinam are listed in Table 1 along with their fold enrichment in mecillinam versus the no drug control condition. The complete set is listed in S1 Table. Representative Tn-Seq profiles of several of the identified resistance loci are shown in Fig 1. As an indication that the analysis was working as expected, several known mecillinam resistance loci were clearly identified, including pbpA, rodA, mreBCD, and slt [4,5,12] (Table 1, Fig 1). Many novel alleles were also uncovered, including sspA, galU, and ptsI. In all, 143 different resistance loci were identified.

**Table 1:** Top sixty mecillinam resistance alleles^a^.

rank	gene^b^	fold enrichment^c^	rank	gene^b^	fold enrichment^c^	rank	gene^b^	fold enrichment^c^
**1**	yrfF*	3903	**21**	tusA**	339	**41**	crr**	133
**2**	***fusA***	3080	**22**	***ubiD***	306	**42**	waaP**	129
**3**	***ileS***	2106	**23**	***mtn***	234	**43**	arcA**	121
**4**	rnt**	1317	**24**	galU**	225	**44**	***rlmH***	120
**5**	ubiE*	1079	**25**	cydB**	220	**45**	crp*	116
**6**	sspA**	1054	**26**	ispA*	219	**46**	***yigP***	104
**7**	tufA**	836	**27**	***rsfS***	207	**47**	rfaH**	103
**8**	***gmhB***	831	**28**	mreB*	194	**48**	***pspE***	98
**9**	mrdA*	814	**29**	***waaF***	190	**49**	***rpoN***	88
**10**	mreC*	750	**30**	***tusD***	186	**50**	sapB**	77
**11**	***rnhA***	744	**31**	***mnmA***	181	**51**	***yheO***	69
**12**	slt*	712	**32**	lpp**	177	**52**	***lipB***	63
**13**	mrdB*	629	**33**	***tusC***	175	**53**	***yaaY***	60
**14**	ybeD**	543	**34**	***ptsI***	167	**54**	gcvR**	59
**15**	***cydA***	518	**35**	iscS**	161	**55**	***ackA***	59
**16**	efp**	476	**36**	cydD**	154	**56**	nlpD**	59
**17**	prc**	464	**37**	ychF**	152	**57**	***yadD***	58
**18**	ubiX*	440	**38**	***aceF***	144	**58**	***lpd***	58
**19**	***ratA***	406	**39**	***hns***	142	**59**	***waaG***	57
**20**	***tusB***	361	**40**	cysE*	141	**60**	aroK*	55

^a^ Top sixty resistance alleles from 10 μg/ml mecillinam plates.

^b^ Genes highlighted by *and ** indicate previously identified or confirmed new mecillinam resistance loci, respectively.

^c^ Fold enrichment was calculated as total transposon insertion reads for a given gene from mecillinam-treated samples divided by the equivalent number of reads from untreated samples.

**Fig 2:**
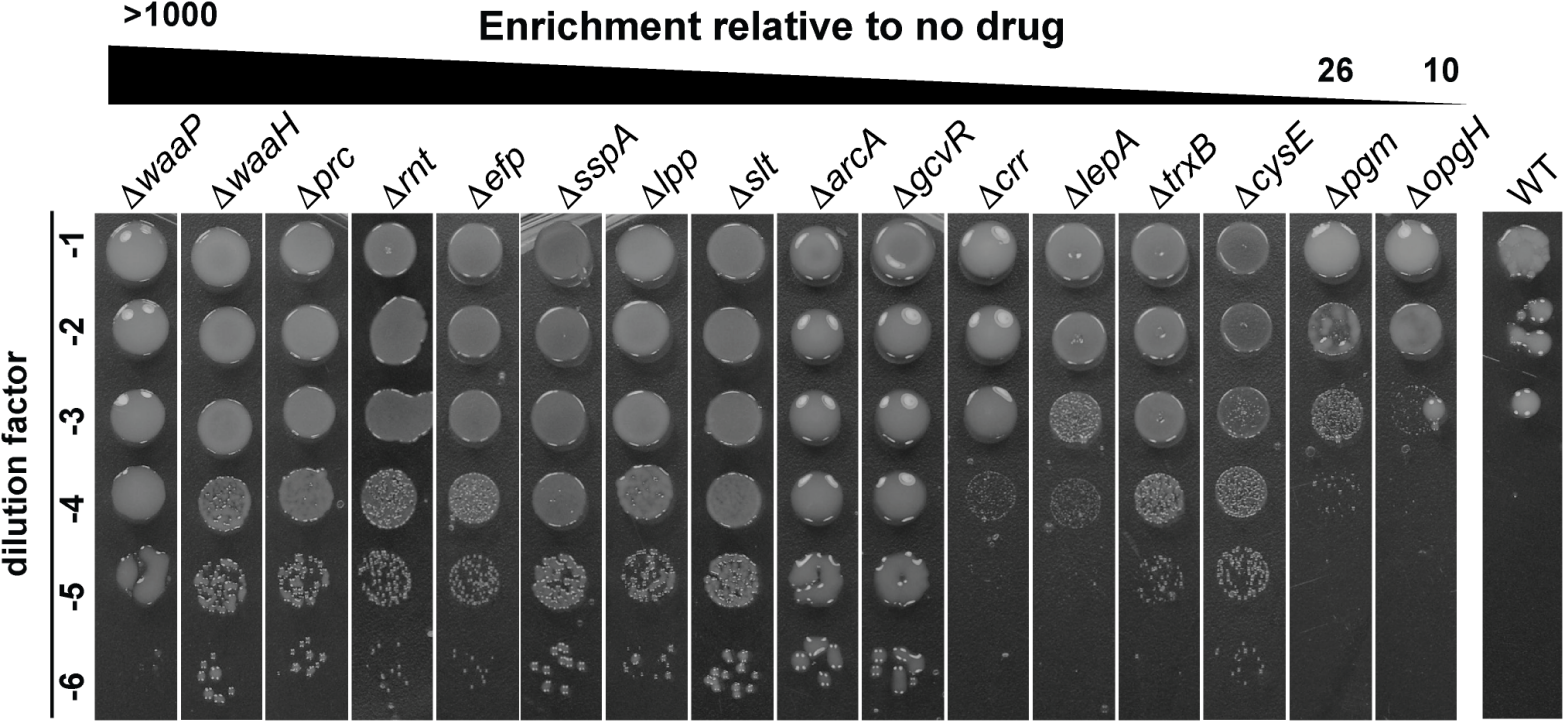
Validation of the mecillinam resistance loci identified by Tn-Seq. Cells of MG1655/pTB63 [WT/ftsZ^up^] or its derivatives with the indicated deletion alleles were grown overnight in LB medium. The resulting cultures were normalized to an OD_600_ of 1.0, serially diluted, and 5 μl of each dilution was spotted on LB agar supplemented with 1 μg/ml mecillinam. Plates were grown for 32 hours at 30^°^C and photographed. Strains were ordered in the figure according to the enrichment ratio of total transposons mapped in each gene in LB with mecillinam relative to the no drug control.

To confirm that inactivation of the identified genes confers mecillinam resistance, relevant deletion-insertion mutants from the Keio collection [27] were transduced into the MG1655/pTB63 background and their mecillinam resistance was assessed. Loci corresponding to a range of different enrichment levels in the Tn-Seq analysis were chosen for validation. Overall, the level of enrichment observed for transposon insertions in a given gene roughly correlated with the degree of mecillinam resistance displayed by the corresponding deletion-insertion mutant (Fig 2). Inactivation of all genes with an enrichment value greater than 26-fold by Tn-Seq provided a clear confirmation of resistance (Table 1, Fig 2). Genes with insertions found at lower enrichment values yielded mixed results and typically conferred only partial resistance when inactivated (Table 1, Fig 2). We conclude that the Tn-Seq analysis has faithfully identified the majority of, if not the complete, mecillinam "resistome" of E. coli.

### Stress response dependence of the mecillinam resistome

**Fig 3:**
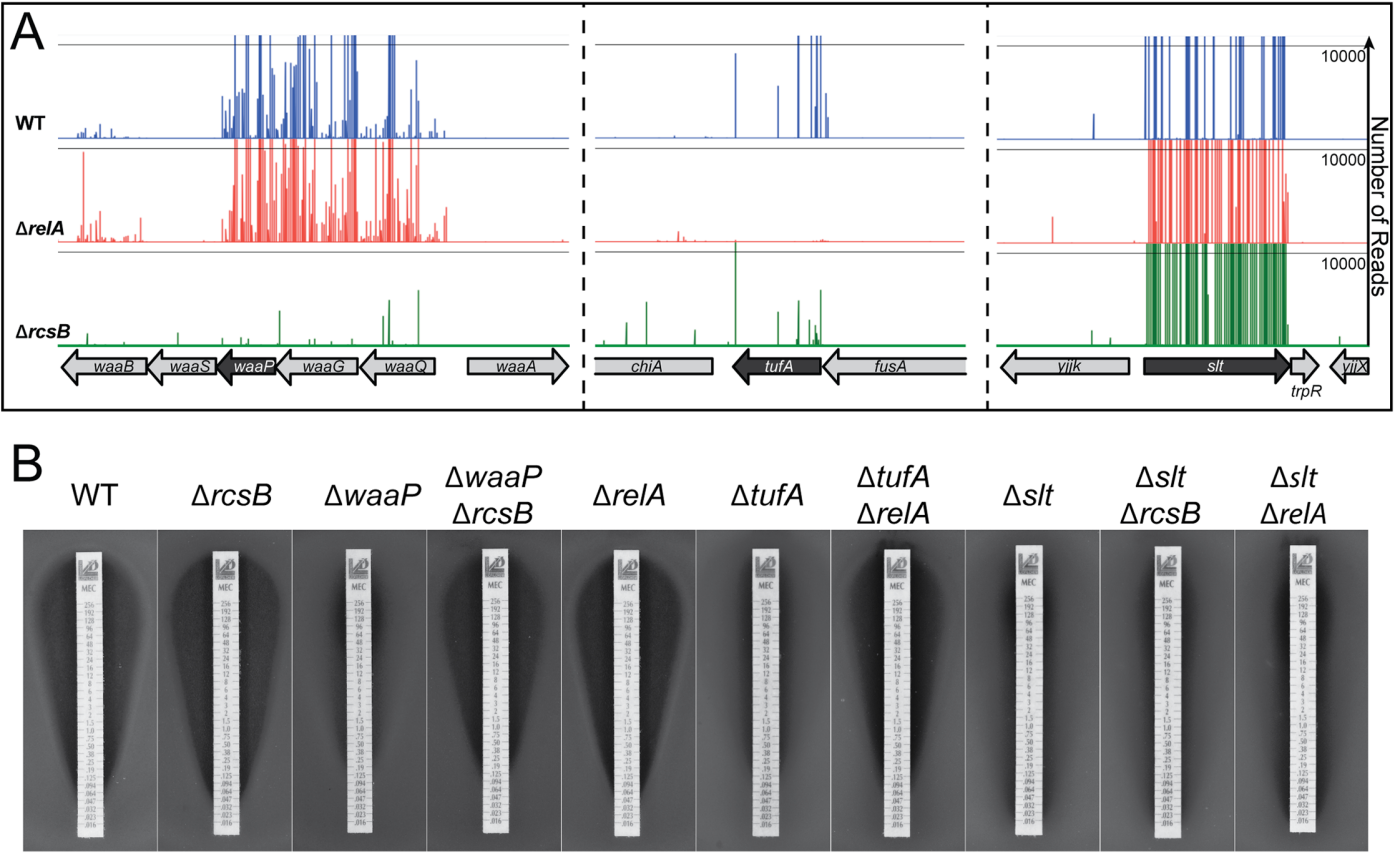
Identification of stress response-dependent mecillinam-resistance alleles. A. Shown are Tn-Seq profiles from transposon libraries prepared in MG1655/pTB63 [WT/ftsZ^up^] and its Δ*r*elA and Δ*rcsB* derivatives as indicated. Profiles are from samples grown on LB supplemented with 10 μg/ml mecillinam. Representative profiles identifying Rcs-dependent (left), RelA-dependent (middle), and stress-response independent (right) loci are shown. B. Stress response-dependence of resistance was confirmed by plating lawns of MG1655/pTB63 [WT/ftsZ^up^] and its indicated derivatives on soft agar. Zones of growth inhibition caused by test strips impregnated with a concentration gradient of mecillinam were then assessed. Lawns were grown at 30^°^C for 18 hours before being photographed. A complete list of resistance loci for the stress response mutant strains and their enrichment ratios (comparing total reads for all insertions with and without drug) is given in S2 Table.

Induction of the stringent response is known to confer mecillinam resistance, and the Rcs envelope stress response pathway has been implicated in the protection of cells from beta-lactam stress [6,20]. Consistent with these findings, a number of loci identified in the Tn-Seq analysis have previously been associated with constitutive production of guanosine tetraphosphate (ppGpp) to induce the stringent response (e.g. *tufA*) [28] or constitutive Rcs activation (e.g. *waaG*) [29]. To identify loci that require induction of either the stringent response or Rcs to confer mecillinam resistance, the Tn-Seq analysis was repeated in either a Δ*relA* or Δ*rcsB* background, respectively. RelA is the major ppGpp synthase in *E*. *coli* [30], and RcsB is the response regulator required to modulate the expression of Rcs-responsive genes [29]. When transposon libraries generated in the Δ*relA* or Δ*rcsB* backgrounds were plated on mecillinam agar, survivors arose at a frequency of 0.5–1 x 10^−3^ and 4–6 x 10^−4^, respectively. The reduced level of survivors in each case relative to WT cells, indicates that many loci identified in the original Tn-Seq analysis are stress response-dependent for resistance. **Table 2**lists the RelA- and RcsB-dependent resistance loci, and **Table 3**lists loci found to be stress response-independent. Full lists of resistance loci identified in the mutant backgrounds are given in **S2 Table**. Representative Tn-Seq profiles for each stress response-dependent/independent gene class are shown in **Fig 3A**. As expected, the RelA-dependent alleles are enriched for genes implicated in translation elongation, tRNA modification, or amino acid metabolism (**Table 2**), indicating that they likely induce ppGpp production when they are inactivated due to adverse effects on protein synthesis. Similarly, many of the RcsB-dependent resistance loci are genes associated with cell envelope biogenesis (**Table 2**), defects in which are among the primary signals that result in Rcs activation.

**Table 2:** Stress response-dependent resistance loci.

**RcsB-dependent loci**^**a**^	**Function**	**RcsB-dependent loci**^**a**^	**Function**
***aceE***	energy metabolism	***ptsI***	carbohydrate transport
***aceF***	energy metabolism	***qseC***	quorum sensing histidine kinase
***ackA***	energy metabolism	***ratA***	ribosome associated toxin
***arcA***	global transcription regulation	***rfaH***	LPS core synthesis
***crr***	central metabolism regulation	***rlmH***	rRNA processing
***galU***	UDP-glucose synthesis	***rsfS***	ribsome maturation and modification
***gmhB***	LPS core synthesis	***sapB***	peptide transport
***hldE***	LPS core synthesis	***sapC***	peptide transport
***icd***	energy metabolism	***sapD***	peptide transport
***iscA***	chaperone for Fe-S clusters	***trkA***	potassium transport
***ispA***	uniquinone biosynthesis	***ubiA***	uniquinone biosynthesis
***lipB***	lipoate biosynthesis	***ubiD***	uniquinone biosynthesis
***lpd***	energy metabolism	***ubiE***	uniquinone biosynthesis
***lpp***	murein lipoprotein	***waaF***	LPS core synthesis
***mtn***	amino acid biosynthesis	***waaP***	LPS core synthesis
***pta***	energy metabolism	***ychF***	ribosome associated protein
***ptsH***	carbohydrate transport	***yigP***	uniquinone biosynthesis
**RelA-dependent loci**^**b**^	**Function**	**RelA-dependent loci**^**b**^	**Function**
***crp***	central metabolism regulation	***proQ***	RNA chaperone
***cysE***	amino acid biosynthesis	***purA***	purine nucleotide biosynthesis
***efp***	protein translation	***sspA***	global transcriptional regulaton
***hns***	global transcriptional regulation	***tufA***	protein translation
***ileS***	tRNA processing	***tusA***	tRNA processing
***nlpI***	lipoprotein adaptor, cell division	***tusB***	tRNA processing

^a^ Alleles designated as RcsB-dependent were identified as mecillinam resistance loci in wild-type cells but not in the Δ*rcsB* background.

^b^ Alleles designated as RelA-dependent were identified as mecillinam resistance loci in wild-type cells but not in the Δ*relA* background.

**Table 3:** Stress response-independent resistance loci.

Gene ^a^	Function	Gene ^a^	Function
***aroK***	amino acid biosynthesis	***mreC***	cell wall synthesis
***cyaA***	central metabolism regulation	***prc***	protease
***cydA***	electron transport	***rnt***	RNA degradation
***cydB***	electron transport	***rodZ***	cell wall synthesis
***cydD***	electron transport	***slt***	cell wall synthesis
***hscA***	chaperone for Fe-S clusters	***tusC***	tRNA processing
***iscS***	tRNA processing	***tusD***	tRNA processing
***mnmA***	tRNA processing	***tusE***	tRNA processing
***mrdA***	cell wall synthesis	***ubiX***	uniquinone biosynthesis
***mrdB***	cell wall synthesis	***yafN***	antitoxin
***mreB***	cell wall synthesis	***ybeD***	unknown

^a^ Alleles designated as stress response-independent were identified as mecillinam resistance loci in wild-type, Δ*rcsB*, and Δ*relA* cells.

To confirm the RelA- or RcsB-dependence of mecillinam resistance for several representative mutants, we plated lawns of the mutants and assessed mecillinam killing using test strips impregnated with a concentration gradient of mecillinam. Resistance due to inactivation of the LPS biogenesis factor WaaP was found to be RcsB-dependent in the Tn-Seq analysis (**Fig 3A**). Consistent with this analysis, the single Δ*waaP* mutant displayed resistance in the test-strip assay, whereas sensitivity was restored in the double Δ*waaP* Δ*rcsB* derivative (**Fig 3B**). Similarly, inactivation of *tufA* encoding translation elongation factor EF-Tu promoted RelA-dependent mecillinam resistance in the Tn-Seq analysis, and this result was confirmed using the test strips (**Fig 3B**). Finally, as expected, blocking the futile cycle of PG synthesis and degradation by Slt inactivation showed stress response-independent mecillinam resistance in the Tn-Seq profiles (**Fig 3A**). This independence was confirmed in the test strip assay in which the Δ*slt* strain showed similar levels of resistance whether or not it possessed a functional RelA or Rcs response (**Fig 3B**). Several other mutants in each category displayed the expected phenotype in the test strip assay based on their behavior in the Tn-Seq analysis in the various mutant backgrounds. We therefore conclude that the analysis correctly defined the stress response-dependence of most mecillinam resistance loci. Further study of stress response induction in mutants defective for loci identified as RelA- or RcsB-dependent for resistance may reveal new information about the precise signals stimulating these important global regulatory systems.

### Effect of the Rcs and stringent responses on cell wall synthesis and the futile cycle

**Fig 4:**
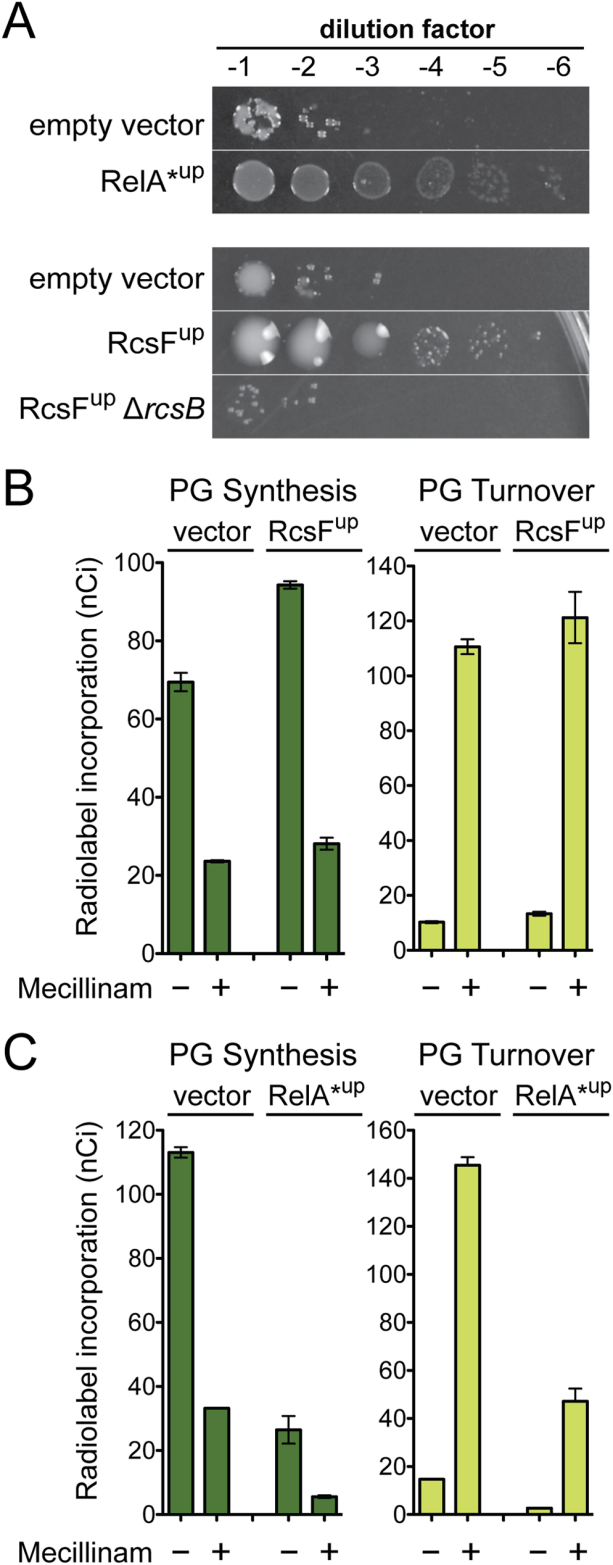
Effect of Rcs and stringent responses on mecillinam toxicity and its induction of cell wall synthesis and turnover. A. Cells of MG1655/pTB63 [WT/ftsZ^up^] and its indicated derivatives harboring chromosomally integrated expression constructs attλGL65 [P_ara_::relA*], attλGL68 [P_tac_::rcsF], or empty vector controls were grown, serially diluted, and plated as in Fig 2. Here, the LB agar was supplemented with mecillinam (1 μg/ml) and the appropriate inducer (top, 0.2% arabinose; bottom 1 mM IPTG). B-C. Radiolabeling assays were performed with strain TU278(attHKHC859) harboring the expression constructs from panel A to measure the incorporation of [^3^H]-mDAP into peptidoglycan and PG turnover products with or without mecillinam (10 μg/ml) treatment. Note that cells are inhibited for cell division such that radiolabeling results reflect cell elongation activity only. Results are the average of three independent experiments with the error bars representing the standard error of the mean. See text and Methods and Materials for assay details.

We have previously shown that beta-lactams inhibit PBPs to cause the formation of un-crosslinked glycans that are rapidly degraded by Slt [12]. For mecillinam, the resulting futile-cycle of PG synthesis and degradation by the drug-targeted Rod complex contributes significantly to its killing activity. We were interested in determining how the Rcs and stringent responses might affect the mecillinam-induced futile cycle to promote survival. Do they antagonize Rod system activity to limit nascent PG degradation, or do the changes in gene expression instead allow cells to cope with the toxic side effects of the futile cycle? To investigate these possibilities, we generated constructs that overexpress either *relA* or *rcsF* to stimulate ppGpp production or the Rcs response, respectively. RcsF is an outer membrane lipoprotein that functions as an inducer of the Rcs system when it is improperly localized in the envelope [31–33]. The RelA produced from our construct was a truncated form (RelA*) predicted to be hyperactive for ppGpp production [30]. As expected from prior genetic analyses and the results presented above, both factors were sufficient to promote mecillinam resistance when overproduced to induce their respective responses (**Fig 4A**).

Mecillinam-induced PG turnover was monitored using a previously established radiolabeling protocol [12,34]. In this assay, cells were first blocked for divisome function by production of the FtsZ antagonist SulA. Division inhibition focuses the PG synthesis measurements on cell elongation activity. Cells with or without drug treatment were then pulse labeled with the radiolabeled PG precursor [^3^H]-diaminopimelic acid ([^3^H]-DAP). After only an additional 1/10th of a generation of growth, the distribution of the label between the PG matrix and PG turnover products was determined. In the absence of mecillinam, cells harboring the vector control incorporated most of the label into the PG matrix with very little material being converted to degradation products (**Fig 4B**). As observed previously [12,34], mecillinam treatment resulted in the conversion of most of the PG material into turnover products (**Fig 4B**). Prior studies have shown that this induction of turnover is blocked by the MreB antagonist A22, indicating that the synthesis and degradation detected is carried out by the Rod system [12]. Overproduction of RcsF did not significantly affect the level of mecillinam induced turnover compared to the vector control (**Fig 4B**). For RelA*-producing cells, the overall levels of PG synthesis and turnover were lower due to the reduced growth rate imposed by ppGpp accumulation. However, the relative level of turnover to synthesis in mecillinam-treated cells was similar to cells with the empty vector control (**Fig 4C**). We conclude that the resistance promoted by the induction of the Rcs and stringent responses is not due to an inhibition of the futile cycle. Instead, the Rcs response is most likely helping cells deal with the consequences of the futile cycle by modulating the expression of genes involved in cell envelope biogenesis. The effects of the stringent response, on the other hand, most likely stem from a reduced growth rate, which is expected to generally limit PG synthesis and therefore may allow cells to cope better with an active futile cycle.

### Overproduction of PG endopeptidases promotes mecillinam resistance

**Fig 5:**
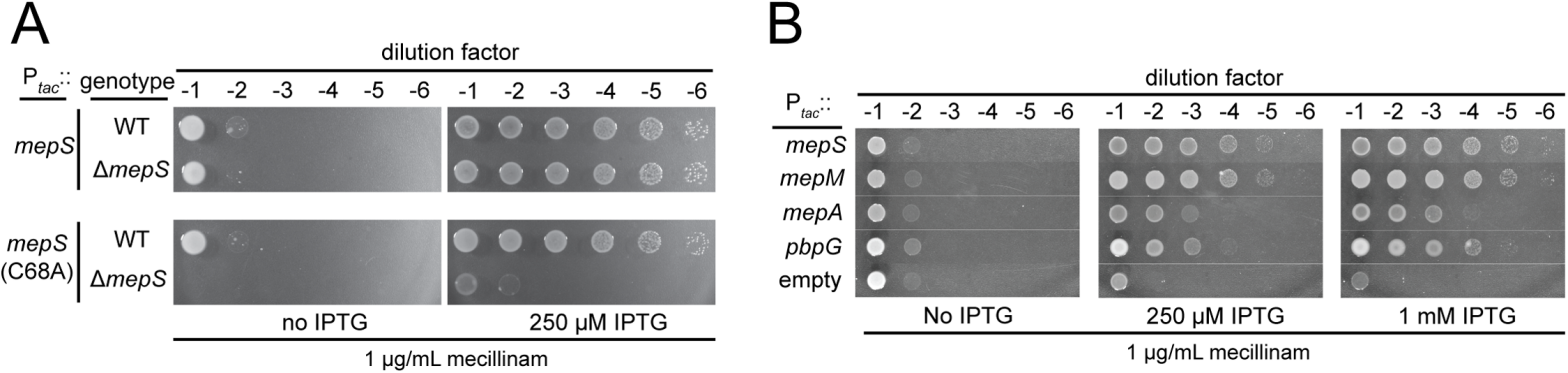
Endopeptidase overproduction promotes mecillinam resistance. A. Cells of MG1655/pTB63 [WT/ftsZ^up^] and its indicated derivatives harboring chromosomally integrated expression constructs attλGL66 [P_tac_::mepS] or attλGL67 [P_tac_::mepS(C68S)] were grown, serially diluted, and plated as in Fig 2. Agar was supplemented with 1 μg/ml mecillinam with or without IPTG as indicated. B. Cells of MG1655/pTB63 [WT/ftsZ^up^] harboring multicopy plasmids pTK2 [P_tac_::mepS], pTK1 [P_tac_::mepM], pTK4 [P_tac_::mepA], pTKD4 [P_tac_::pbpG], and pHC800 [P_tac_::empty] were grown, serially diluted, and plated as in Fig 2. Agar was supplemented with 1 μg/ml mecillinam with or without IPTG as indicated.

Stress response-independent mecillinam resistance loci included genes coding for components of the Rod system [*mreB*, *mreC*, *rodZ*, *mrdA* (encoding PBP2), and *mrdB* (encoding RodA)] and *slt*, which encodes the LT responsible for mecillinam-induced PG turnover. We therefore suspected that other genes included in this class may also encode factors that directly or indirectly alter PG biogenesis. We were particularly interested in *prc* (**Fig 2**) given that it encodes a protease recently shown to be involved in the turnover of MepS (Spr) [22], a PG endopeptidases implicated in PG matrix expansion [21]. This observation was intriguing because inactivation of MepS has the opposite phenotype. It was found to result in mecillinam hypersensitivity in a high-throughput chemical genomic screen of the *E*. *coli* Keio collection [35]. Thus, the mechanism by which Prc inactivation suppresses mecillinam toxicity might in part be through the overproduction of MepS. Indeed, overexpression of *mepS* was capable of promoting mecillinam resistance (**Fig 5**), and MepS is required for mecillinam resistance in Δ*prc* cells (**S1 Fig**).

To determine if suppression by *mepS* overexpression required the endopeptidase activity of MepS, we generated an overexpression vector encoding MepS(C68A), in which the active site Cys was replaced by Ala [21]. Surprisingly, overproduction of MepS(C68A) was also capable of promoting growth in the presence of mecillinam (**Fig 5A**). One possible explanation for this result is that MepS catalytic activity is not required for mecillinam suppression. Alternatively, the overproduction of MepS(C68A) in otherwise wild-type cells might overwhelm the Prc protease and thereby stabilize and increase the levels of the natively produced MepS(WT) protein. In support of the latter possibility, overproduction of MepS(C68A) was unable to suppress mecillinam toxicity in a strain deleted for the native copy of the *mepS* gene (**Fig 5A**). To determine if the suppression activity was specific to MepS, we tested the effect of overproduction of other *E*. *coli* PG endopeptidases on mecillinam killing activity. Remarkably, overproduction of several additional endopeptidases was capable of promoting growth on mecillinam agar: MepM (YebA) and MepA, which are LAS-family metallo-endopeptidases, and PbpG (PBP7), which is a cPBP-type endopeptidase [36] (**Fig 5B**). Both of these protein families are distinct from the NlpC/P60 family to which MepS belongs [36]. As with MepS, a variant of MepM with substitutions predicted to inactivate endopeptidase activity was incapable of promoting mecillinam resistance when overproduced (**S2 Fig**). Based on this series of experiments, we conclude that elevated PG endopeptidase activity promotes survival upon mecillinam treatment. This observation is surprising because cell wall hydrolase activity is typically thought to promote the lethal and lytic effects of beta-lactam antibiotics, not to counteract them [23].

### MepS overproduction suppresses the mecillinam induced futile cycle by boosting PG synthesis

**Fig 6:**
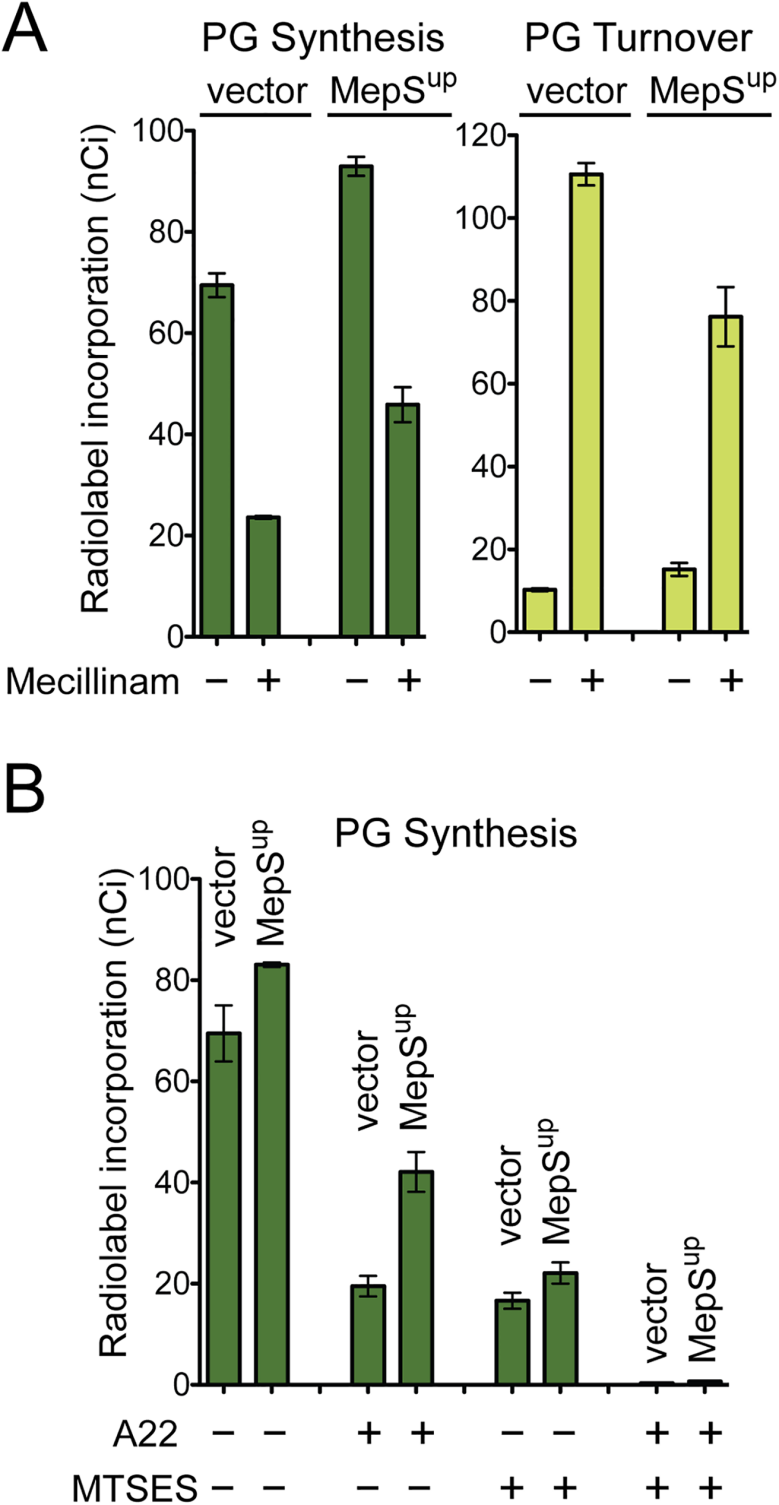
MepS overproduction enhances PG synthesis and suppresses mecillinam-induced PG turnover. A. Cells of TU278(attHKHC859) harboring integrated expression constructs attλGL70 [P_tac_::empty] or attλGL66 [P_tac_::mepS] were used in physiological radiolabeling assays to measure the incorporation of [^3^H]-mDAP into peptidoglycan and PG turnover products with or without mecillinam (10 μg/ml) treatment. The RcsF overproduction experiments from Fig 4 were performed together with the MepS experiments shown here. Because it served as a control for both experiments the empty vector data from panel 4B was repeated here. B. [^3^H]-mDAP incorporation into the PG matrix was followed in cells of HC533(attHKHC859) harboring the same expression constructs as in A. This strain encodes the MTSES-sensitive variant of PBP1b (^MS^PBP1b) as its only aPBP. Label incorporation was monitored in cells inhibited for division following treatment with A22 and/or MTSES as indicated. A22 disrupts Rod system function such that only aPBPs are functional for PG polymerization. MTSES blocks ^MS^PBP1b activity such that PG polymerization is restricted to RodA functioning within the Rod system. Dual treatment with A22 and MTSES blocks all polymerization. Note that cells are inhibited for cell division such that radiolabeling results reflect cell elongation activity only. Results are the average of three independent experiments with the error bars representing the standard error of the mean. See text and Methods and Materials for assay details.

To investigate the mechanism by which endopeptidase overproduction suppresses mecillinam toxicity, we monitored the effect of MepS overproduction on the mecillinam-induced futile cycle of cell wall synthesis and degradation. Notably, the incorporation of label into the PG matrix was increased in mecillinam-treated cells overproducing MepS relative to the vector control, and this increase was accompanied by a decrease in the level of PG turnover (**Fig 6A**). Thus, increased MepS endopeptidase activity appears to promote survival by limiting the mecillinam-induced futile cycle. A possible explanation for this activity is that MepS overproduction stimulates the activity of PG synthases functioning outside of the Rod complex thereby redirecting PG precursors from the crippled Rod complex to a functional synthetic machinery.

Because cell division was inhibited in the labeled cells, the aPBPs are the functional machinery to which precursors are redirected in cells overproducing MepS. In support of this possibility, we recently showed that the SEDS protein RodA serves as the PG polymerase in the Rod system [8] and that the aPBP synthases can operate independently of the cytoskeletally-organized PG synthesis complexes [9]. To directly test the effect of MepS overproduction on aPBP activity, we took advantage of an in vivo PG labeling system in which the PG biogenesis activity of the Rod system or the aPBPs can be independently measured. For these assays, we use a strain producing a modified PBP1b, referred to as ^MS^PBP1b, as its only aPBP. This variant has a Ser247Cys substitution in its GT domain rendering it sensitive to inhibition by treatment with the cysteine-reactive reagent MTSES (2-sulfonatoethyl methanethiosulfonate) [9]. When cells of this strain are inhibited for cell division, total [^3^H]-DAP incorporation into the PG matrix represents a combination of the activities of the Rod system and ^MS^PBP1b [9]. Upon treatment with the Rod system inhibitor A22, the remaining level of [^3^H]-DAP incorporation reflects the activity of ^MS^PBP1b, whereas the level of PG synthesis detected in MTSES-treated cells is a measure of Rod system activity [9]. Importantly, and as expected based on this line of reasoning, co-treatment with A22 and MTSES completely inhibits all detectable [^3^H]-DAP incorporation [9] (**Fig 6B**).

Overproduction of MepS resulted in a small but reproducible increase in [^3^H]-DAP incorporation into the PG matrix in untreated cells relative to those harboring the vector control (**Fig 6A and 6B**). Strikingly, however, PG synthesis following A22 treatment more than doubled in cells overproducing MepS, suggesting that elevated endopeptidase activity greatly enhances PG synthesis by ^MS^PBP1b. Accordingly, this elevated level of incorporation was completely inhibited by simultaneous treatment with A22 and MTSES (**Fig 6B**). The observed activation appeared to be specific for aPBP synthase function as MepS overproduction did not enhance label incorporation in cells treated with MTSES alone where PG is primarily being synthesized by the Rod system (**Fig 6B**). The labeling results suggest that elevated PG endopeptidase activity can stimulate PG synthesis by the aPBPs either by increasing aPBP levels or their activity. The observation that overproduction of PBP1b or a PBP1b variant [PBP1b(E313D)] that functions independently of its activator LpoB [37] did not promote mecillinam resistance favors the latter possibility.

**Fig 7:**
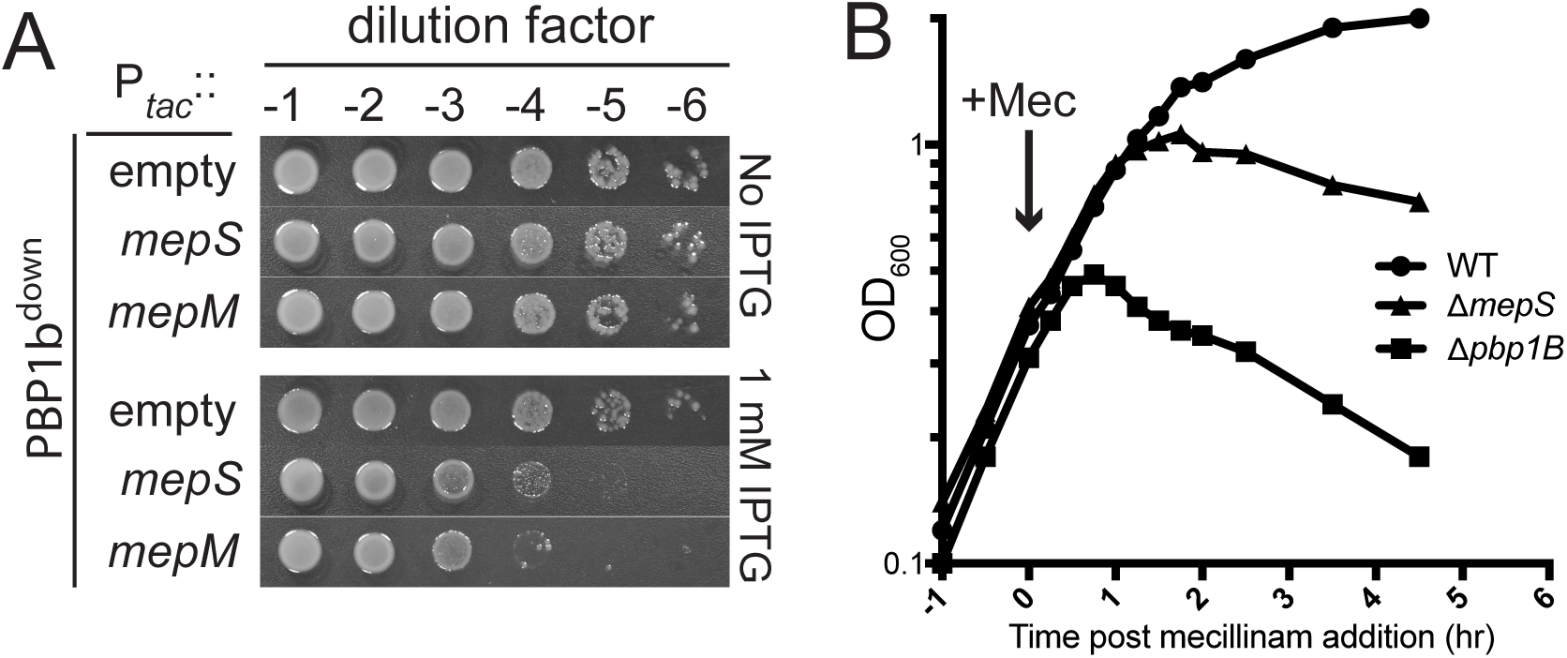
Phenotypic connections between PBP1b and MepS. A. Cells of HC509 [P_ara_::ponB (encoding PBP1b)] harboring plasmids pHC800 [P_tac_::empty], pTK2 [P_tac_::mepS], or pTK1 [P_tac_::mepM] as indicated were grown overnight in M9 minimal medium containing 0.2% arabinose, washed in LB containing 0.2% glucose, serially diluted, and plated on LB agar (no drug) supplemented with 0.2% glucose to repress PBP1b production and IPTG as indicated to induce expression of the endopeptidase encoding genes. B. Cells of MG1655 [WT], GL67 [Δ*ponB*], or GL68 [Δ*mepS*] were diluted in LB broth to an OD_600_ of 0.05 and grown at 30^°^C. When cultures reached an OD_600_ of approximately 0.3, mecillinam was added at 0.06 μg/ml (0.25 x MIC of WT cells). Growth was continued at 30^°^C and monitored by regular measurements of culture OD_600_.

Additional support for the connection between endopeptidase function and aPBP activity came from two observations. The first is that mutants defective for PBP1b are sensitive to the overproduction of MepS and other endopeptidases (**Fig 7A**), a result consistent with this PBP being required to add PG material to the voids in the matrix created by the cleavage of crosslinks. Secondly, mutants defective for MepS and PBP1b-inactivated cells respond similarly to mecillinam challenge (**Fig 7B**). Wild-type cells grow for some time following mecillinam addition and form large spheres before lysing. Because the Rod system is malfunctioning and these cells are not dividing, expansion of the spherical cells prior to lysis is most likely mediated by the aPBPs functioning outside of the cytoskeletal systems [9]. Consistent with this idea, mutants lacking PBP1b lyse relatively rapidly after mecillinam treatment [38] (**Fig 7B**). Cells defective for MepS also lyse following mecillinam treatment, suggesting that aPBP activity not optimal in these cells. The reason they do not lyse as quickly as cells inactivated for PBP1b is probably due to partial redundancy with other endopeptidases in the cell [21] (**Fig 5**). Based on the combination of genetic and radiolabeling results, we conclude that PG endopeptidases are not only required for PG synthesis, their activity also appears to be limiting for PG synthesis by the aPBPs (see Discussion).

## Discussion

In addition to serving as powerful therapeutics, beta-lactams have been useful probes for uncovering the mechanisms underlying the process of cell wall biogenesis in bacteria. Here, we present the first comprehensive genetic analysis of mecillinam resistance in *E*. *coli*. Using Tn-Seq, we simultaneously mapped nearly all loci in the genome where gene disruption by transposon insertion promotes survival upon mecillinam challenge. Moreover, we performed the analysis in genetic backgrounds defective for stress responses known to confer mecillinam resistance when they are induced. Thus, we were able to classify resistance loci according to their stress response dependence, identifying those alleles that most likely promote resistance by activating a stress response and those that confer resistance independent of the responses. We reasoned that many of these stress response-independent alleles are likely to provide mecillinam resistance by directly affecting cell wall biogenesis. Accordingly, this class includes mutants inactivated for components of the Rod system and the cell wall cleaving enzyme Slt known to be required for the futile cycle of cell wall synthesis and degradation observed following mecillinam treatment. Another stress response-independent resistance allele, *prc*, led us to the conclusion that the cleavage of cell wall crosslinks by PG endopeptidases results in the activation of PG synthesis by the aPBPs.

Because the cell wall matrix is a continuous molecular network surrounding the cell, it has long been understood that cleavage of bonds in the matrix should be required for the insertion of newly synthesized PG into the wall and the expansion of the cell surface during growth [24]. However, it was only recently that candidate “space-maker” enzymes required for PG matrix expansion were identified [21,39,40]. In *E*. *coli*, these enzymes are the PG endopeptidases MepS (Spr) and MepM (YebA) [21]. Neither enzyme is essential individually, but cells lacking both endopeptidases are inviable on rich medium. Cells depleted of MepS in the absence of MepM stop elongating and eventually lyse [21]. They also show reduced incorporation of radiolabeled PG precursors into the matrix [21]. Thus, it has been established that MepS and MepM are required for growth and cell wall biogenesis, which is consistent with a “space maker” function. What has remained unclear is how endopeptidase activity is coordinated with cell wall synthase function and whether or not crosslink cleavage by the endopeptidases can precede cell wall synthesis (i.e. can cleavage promote synthesis). Our findings address these important outstanding issues.

Our window into the role of endopeptidases in PG synthesis by the aPBPs came from the observation that the overproduction of representative enzymes from three different endopeptidase families promoted resistance to the beta-lactam mecillinam. This was a surprising finding because cell wall hydrolase activity is typically thought to promote the lethal and lytic effects of beta-lactam antibiotics, not to counteract them [23]. We hypothesized that the increased cleavage of cell wall crosslinks was promoting mecillinam resistance by activating cell wall synthase activity outside of the Rod complex. This activation would effectively redirect cell wall precursors away from the futile cycle of synthesis and degradation promoted by the mecillinam-targeted Rod system, and thereby dampen its toxic effects. Consistent with this idea, MepS overproduction was shown to increase productive PG synthesis in mecillinam-treated cells and to reduce the turnover of nascent PG material. Furthermore, radiolabeling studies monitoring the activity of either the Rod system or the aPBPs showed that MepS overproduction specifically enhanced PG biogenesis by the aPBP enzymes.

Beta-lactam tolerance via the formation of stable and viable spherical cells was recently observed in several gram-negative bacteria [41]. Interestingly, the endopeptidase ShyA was shown to be required for the formation of the tolerant spheres in *Vibrio cholerae*. Also, overexpression of ShyA in enterohemorrhagic *E*. *coli* (EHEC) strain EDL933 promoted a mild enhancement of survival following meropenem treatment [41]. Although the mechanism of beta-lactam tolerance in these systems remains to be determined, the phenomena suggest that the endopeptidase-mediated enhancement of aPBP activity we observe here may be a common mechanism promoting PG synthesis in gram-negative bacteria.

The coordination of PG synthase activity with the space making enzymes is commonly believed to be mediated by the formation of a multi-enzyme complex that includes both PG synthase and PG hydrolase activities [1,24,42]. Although many protein-protein interactions involving PG synthases have been detected, intact complexes have not been isolated [1]. Also, few of the detected interactions involve PG hydrolases, and the physiological relevance of the vast majority of detected interactions remain undetermined [43–46]. Thus, despite being frequently proposed and discussed, evidence in support of multi-enzyme complexes between PG synthases and hydrolases functioning in vivo is limited.

Our results support an alternative model in which PG hydrolases can stimulate PG synthase function without a direct protein-protein interaction. Overproduction of catalytically inactive MepS(C68A) remained capable of stimulating mecillinam resistance provided cells also encoded native functional MepS. We interpret this result to indicate that the overproduced MepS(C68A) overwhelms the Prc protease that degrades MepS, thus elevating the levels of the active protein. In this scenario, the periplasm is flooded with excess MepS(C68A) that would presumably occupy the binding sites of most MepS interacting partners. Thus the active MepS in this context is unlikely to be functioning in complex with a cell wall synthase to promote productive PG assembly during mecillinam challenge. Similarly, the fact that the overproduction of three different endopeptidases each from a distinct protein family are all capable of promoting mecillinam resistance argues against a specific protein-protein interaction with a PG synthase binding partner being involved.

How might crosslink cleavage and PG synthase activity be coupled if not via a direct protein-protein interaction? One attractive possibility is via the regulation of the aPBPs by their cognate outer membrane lipoproteins. Several years ago, it was discovered that the *E*. *coli* aPBPs, PBP1a and PBP1b, each require a cognate outer membrane lipoprotein activator, LpoA and LpoB, respectively for their in vivo function [47,48]. It was also shown that these Lpo factors can stimulate the PG synthase activity of their cognate aPBP in vitro [37,47–49]. Based on the trans-envelope nature of the aPBP-Lpo complexes, it was previously proposed that the Lpo-PBP interaction might function as a “sensor” for the detection of loosely crosslinked areas in the PG matrix [1]. According to the model, these areas of the matrix are where an Lpo protein and partner PBP could span the matrix from opposite membranes to interact, thus stimulating PG synthesis exactly where it is most needed. Such a mechanism may be at the heart of the stimulation of aPBP activity we observe upon endopeptidase overproduction. In this case, no direct interaction between the aPBP and endopeptidase would be required, rather the aPBP-Lpo complexes would “sense” the cut sites and “fill-in” accordingly.

In conclusion, we have used high-throughput genetic methods to map the mecillinam resistome of *E*. *coli*. Using several different genetic backgrounds for this analysis, we were able to rapidly identify resistance loci that promote resistance without requiring a functional stringent response or Rcs envelope stress response. Characterization of one of these alleles led to a new fundamental understanding of the cell wall biogenesis process: that endopeptidase activity stimulates the activity of aPBP synthases and that the synthases need not work in direct physical contact with the endopeptidases to properly coordinate PG synthesis with cleavage as is commonly believed. Further studies of other loci identified in the mecillinam resistome should shed further light on the mechanism of cell wall biogenesis and how best to target the process for the development of new antibiotics capable of defeating resistance.

## Materials and methods

### Media, bacterial strains, and plasmids

Cells were grown in LB [1% tryptone, 0.5% yeast extract, 0.5% NaCl] or minimal M9 medium [50] supplemented with 0.2% casamino acids and 0.2% maltose. Unless otherwise indicated, antibiotics were used at 25 (chloramphenicol; Cm), 25 (kanamycin; Kan), or 15 (ampicillin; Amp) μg/ml.

The bacterial strains used in this study are listed in S3 Table. All E. coli strains used in the reported experiments are derivatives of MG1655 [51]. Plasmids used in this study are listed in S4 Table. PCR was performed using Q5 polymerase (NEB) for cloning purposes and Taq DNA polymerase (NEB) for diagnostic purposes, both according to the manufacturer’s instructions. Unless otherwise indicated, MG1655 chromosomal DNA was used as the template. Plasmid DNA and PCR fragments were purified using the Zyppy plasmid miniprep kit (Zymo Research) or the Qiaquick PCR purification kit (Qiagen), respectively.

### Transposon mutagenesis and suppressor selection

MG1655/pTB63 or its Δ*relA* or Δ*rcsB* derivatives were mutagenized with the Ez-Tn5 <Kan-2> transposome (Epicentre) as previously described [52]. Mutants were selected on agar for kanamycin resistance at 30°C, yielding libraries ranging from ~100,000 to ~400,000 independent transposon insertions. The mutant libraries were harvested by scraping colonies from the agar surface and suspending them in LB broth. The suspended cells were then plated on LB agar with 0, 1.0, 2.5, or 10 μg/ml mecillinam and incubated at 30°C to isolate mecillinam resistant survivors. The collection of mutants was again harvested by scraping colonies from the agar surface and suspended in LB broth.

### Transposon sequencing

Genomic DNA was extracted from the suspended collection of mutants using the Wizard Genomic DNA Purification Kit (Promega). Tn-seq sequencing libraries were prepared by a modified version of a published protocol [19]. Genomic DNA was fragmented using NEBNext dsDNA Fragmentase (NEB) for 25min at 37°C. Fragmented DNA was purified with 1.8× volume Agentcourt AMPure XP beads (Beckman Coulter, Inc.) and eluded into 32 μl water.

Purified fragmented DNA was then treated with terminal deoxynucleotidyl transferase (TdT; Promega) in a 20 μl reaction with 1 μL 9.5mM dCTP/0.5mM ddCTP, 4 μl 5× TdT reaction buffer and 0.5 μl rTdT at 37°C for 1h, then at 75°C for 20min. TdT-treated DNA was purified with Performa DTR Gel Filtration Cartridge (EdgeBio). Purified, TdT-treated DNA was used as a template in a PCR reaction to amplify the transposon junctions using the Easy-A Hi-Fi Cloning System (Agilent Technologies). The primers used were:

**PolyG-1st-1** 5’-GTGACTGGAGTTCAGACGTGTGCTCTTCCGATCTGGGGGGGGGGGGGGGG-3’
and **Tn5-1st-1** 5’-ACCTGCAGGCATGCAAGCTTCAGGG-3’

A second nested PCR was next performed to further amplify the transposon junctions and append the sequencing barcode. The primers used were generic NEBNext Multiplex Oligos for Illumina (NEB) and:

**Tn5-2nd-1 5’-**AATGATACGGCGACCACCGAGATCTACACTCTTTTCAGGGTTGAGATGTGTATAAGAGA-3’.

The final product was run on a 2% agarose gel, and fragments ranging from 200–500 bp were gel purified using QIAquick Gel Extraction Kit (Qiagen). Libraries were sequenced at the Tufts University Core Facility on a HiSeq 2500 (Illumina) on a 1× 100 single end run.

Reads were mapped to the *E*. *coli* MG1655 genome (NCBI NC_000913), and genes in which reads were overrepresented were identified by calculating the fold change enrichment under mecillinam conditions relative to LB conditions. Genes in which insertions were at least 4-fold enriched in the presence of mecillinam are listed in S1 and S2 Tables. Visual inspection of transposon insertion profiles was performed with the Sanger Artemis Genome Browser and Annotation tool.

### Mecillinam susceptibility testing

Overnight cultures of the strains of interest were normalized for optical density. The were then serially diluted, and 5 μl of each dilution was spotted on LB with or without added mecillinam at a range of concentrations. Alternatively, normalized cultures were mixed with molten soft agar and applied to LB plates as a thin layer. An MIC Test Strip (Liofilchem) was then applied to the soft agar surface, and plates were incubated for 18h before being photographed.

### Cell wall synthesis and turnover measurements

PG synthesis and turnover in beta-lactam-treated *E*. *coli* cells was examined essentially as described previously [9]. TU278(attHKHC859) or HC533(attλHC739), Δ*lysA* Δ*ampD* strains, or their derivatives were grown overnight in M9- glycerol medium supplemented with 0.2% CAA. The overnight culture was diluted to an optical density at 600 nm (OD_600_) of 0.04 in the same medium and grown to an OD_600_ of between 0.26 and 0.3. Divisome formation was then blocked by inducing *sulA* expression for 30 min from a chromosomally integrated P_tac_::*sulA* construct (pHC739) by adding IPTG to 1 mM. After adjusting the culture OD_600_ to 0.3, MTSES (1 mM), A22 (10 μg/ml), and/or mecillinam (10 μg/ml) were added to the final concentrations indicated and cells were incubated for 5 min. Following drug treatment, 1 μCi of [^3^H]-meso-2,6-diaminopimelic acid (mDAP) was added to 1 ml of each drug-treated culture and incubated for 10 min to label the newly synthesized PG and its turnover products. After labelling, cells were pelleted, resuspended in 0.7 ml water, and heated at 90°C for 30 min to extract water-soluble compounds. After hot water extraction, insoluble material was pelleted by ultracentrifugation (200,000 x g for 20 min at 4°C). The resulting supernatant was then removed, lyophilized and resuspended in 0.1% formic acid for HPLC analysis and quantification of turnover products as described previously. To determine the [^3^H]-mDAP incorporated into the PG matrix, the pellet fraction was washed with 0.7 ml buffer A (20 mM Tris-HCl, pH 7.4, 25 mM NaCl) and resuspended in 0.5 ml buffer A containing 0.25 mg lysozyme. The suspensions were incubated overnight at 37°C. Insoluble material was then pelleted by centrifugation (21,000g for 30 min at 4°C) and the resulting supernatant was mixed with 10 ml EcoLite (MP Biomedicals) scintillation fluid and quantified in a Microbeta Trilux 1450 liquid scintillation counter (Perkin-Elmer).

## Supporting information

S1 Fig Inactivation of MepS restores mecillinam sensitivity to Prc-defective cells. Lawns of TB28/pTB63 [WT/*ftsZ*^up^] and its indicated derivatives were plated in soft agar and incubated with mecillinam test strips as in Fig 3. Note that loss of Prc function results in resistance and that sensitivity is restored by MepS inactivation.
(TIF)

S2 Fig MepM lacking catalytic residues is unable to promote mecillinam resistance. Overnight cultures of MG1655/pTB63 with plasmids pTK1 [P_tac_::*mepM*], pTKD8 [P_tac_::*mepM*(SLY)], or pHC800 [P_tac_::*empty*] were serially diluted and spotted on LB agar containing mecillinam and IPTG at the indicated concentrations. The plates were incubated at 30^°^C for 24 hrs and photographed. The *mepM*(SLY) gene encodes *MepM* with residues HLH(393–395) converted to SLY. This substitution mimics the defective LytM active site of the related EnvC protein.
(TIF)

S1 Text Supplementary methods describing plasmid constructions. (DOCX)

S1 Table Transposon insertion enrichment ratios for identified mecillinam resistance loci in WT cells. (XLSX)

S2 Table Transposon insertion enrichment ratios for identified mecillinam resistance loci in Δ*r*elA and ΔrcsB cells. (XLSX)

S3 Table Strains used in this study. (PDF)

S4 Table Plasmids used in this study. (PDF)

## References

[1] Typas A, Banzhaf M, Gross CA, Vollmer W. From the regulation of peptidoglycan synthesis to bacterial growth and morphology. Nat Rev Microbiol. 2012;10: 123–136. doi: 10.1038/nrmicro2677 22203377 10.1038/nrmicro2677 PMC5433867

[2] Tipper DJ, Strominger JL. Mechanism of action of penicillins: a proposal based on their structural similarity to acyl-D-alanyl-D-alanine. Proc Natl Acad Sci USA. 1965;54: 1133–1141. 5219821 10.1073/pnas.54.4.1133 PMC219812

[3] Sauvage E, Kerff F, Terrak M, Ayala JA, Charlier P. The penicillin-binding proteins: structure and role in peptidoglycan biosynthesis. FEMS Microbiol Rev. 2008;32: 234–258. doi: 10.1111/j.1574-6976.2008.00105.x 18266856 10.1111/j.1574-6976.2008.00105.x

[4] Spratt BG. Distinct penicillin binding proteins involved in the division, elongation, and shape of Escherichia coli K12. Proc Natl Acad Sci USA. 1975;72: 2999–3003. 1103132 10.1073/pnas.72.8.2999 PMC432906

[5] Wachi M, Doi M, Tamaki S, Park W, Nakajima-Iijima S, Matsuhashi M. Mutant isolation and molecular cloning of mre genes, which determine cell shape, sensitivity to mecillinam, and amount of penicillin-binding proteins in Escherichia coli. J Bacteriol. 1987;169: 4935–4940. 2822655 10.1128/jb.169.11.4935-4940.1987 PMC213889

[6] Vinella D, D'Ari R, Jaffé A, Bouloc P. Penicillin binding protein 2 is dispensable in Escherichia coli when ppGpp synthesis is induced. EMBO J. 1992;11: 1493–1501. 1563353 10.1002/j.1460-2075.1992.tb05194.x PMC556598

[7] Henriques AO, Glaser P, Piggot PJ, Moran CP. Control of cell shape and elongation by the rodA gene in Bacillus subtilis. Molecular Microbiology. 1998;28: 235–247. 9622350 10.1046/j.1365-2958.1998.00766.x

[8] Meeske AJ, Riley EP, Robins WP, Uehara T, Mekalanos JJ, Kahne D, et al SEDS proteins are a widespread family of bacterial cell wall polymerases. Nature. 2016;537: 634–638. doi: 10.1038/nature19331 27525505 10.1038/nature19331 PMC5161649

[9] Cho H, Wivagg CN, Kapoor M, Barry Z, Rohs PDA, Suh H, et al Bacterial cell wall biogenesis is mediated by SEDS and PBP polymerase families functioning semi-autonomously. Nat Microbiol. 2016;1: 16172 doi: 10.1038/nmicrobiol.2016.172 27643381 10.1038/nmicrobiol.2016.172 PMC5030067

[10] Kruse T, Bork-Jensen J, Gerdes K. The morphogenetic MreBCD proteins of Escherichia coli form an essential membrane-bound complex. Molecular Microbiology. 2004;55: 78–89. doi: 10.1111/j.1365-2958.2004.04367.x 15612918 10.1111/j.1365-2958.2004.04367.x

[11] Bendezú FO, de Boer PAJ. Conditional lethality, division defects, membrane involution, and endocytosis in mre and mrd shape mutants of Escherichia coli. J Bacteriol. 2008;190: 1792–1811. doi: 10.1128/JB.01322-07 17993535 10.1128/JB.01322-07 PMC2258658

[12] Cho H, Uehara T, Bernhardt TG. Beta-lactam antibiotics induce a lethal malfunctioning of the bacterial cell wall synthesis machinery. Cell. 2014;159: 1300–1311. doi: 10.1016/j.cell.2014.11.017 25480295 10.1016/j.cell.2014.11.017 PMC4258230

[13] Costa CS, Antón DN. High-level resistance to mecillinam produced by inactivation of soluble lytic transglycosylase in Salmonella enterica serovar Typhimurium. FEMS Microbiol Lett. 2006;256: 311–317. doi: 10.1111/j.1574-6968.2006.00133.x 16499622 10.1111/j.1574-6968.2006.00133.x

[14] Vinella D, Albrecht C, Cashel M, D'Ari R. Iron limitation induces SpoT-dependent accumulation of ppGpp in Escherichia coli. Molecular Microbiology. 2005;56: 958–970. doi: 10.1111/j.1365-2958.2005.04601.x 15853883 10.1111/j.1365-2958.2005.04601.x

[15] Vinella D, Cashel M, D'Ari R. Selected amplification of the cell division genes ftsQ-ftsA-ftsZ in Escherichia coli. Genetics. 2000;156: 1483–1492. 11102351 10.1093/genetics/156.4.1483 PMC1461353

[16] Vinella D, Gagny B, Joseleau-Petit D, D'Ari R, Cashel M. Mecillinam resistance in Escherichia coli is conferred by loss of a second activity of the AroK protein. J Bacteriol. 1996;178: 3818–3828. 8682786 10.1128/jb.178.13.3818-3828.1996 PMC232642

[17] Vinella D, Joseleau-Petit D, Thévenet D, Bouloc P, D'Ari R. Penicillin-binding protein 2 inactivation in Escherichia coli results in cell division inhibition, which is relieved by FtsZ overexpression. J Bacteriol. 1993;175: 6704–6710. 8407846 10.1128/jb.175.20.6704-6710.1993 PMC206783

[18] Thulin E, Sundqvist M, Andersson DI. Amdinocillin (Mecillinam) resistance mutations in clinical isolates and laboratory-selected mutants of Escherichia coli. Antimicrob Agents Chemother. 2015;59: 1718–1727. doi: 10.1128/AAC.04819-14 25583718 10.1128/AAC.04819-14 PMC4325821

[19] van Opijnen T, Bodi KL, Camilli A. Tn-seq: high-throughput parallel sequencing for fitness and genetic interaction studies in microorganisms. Nat Methods. 2009;6: 767–772. doi: 10.1038/nmeth.1377 19767758 10.1038/nmeth.1377 PMC2957483

[20] Laubacher ME, Ades SE. The Rcs phosphorelay is a cell envelope stress response activated by peptidoglycan stress and contributes to intrinsic antibiotic resistance. J Bacteriol. 2008;190: 2065–2074. doi: 10.1128/JB.01740-07 18192383 10.1128/JB.01740-07 PMC2258881

[21] Singh SK, L S, Amrutha RN, Reddy M. Three redundant murein endopeptidases catalyze an essential cleavage step in peptidoglycan synthesis of Escherichia coli K12. Molecular Microbiology. 2012;86: 1036–1051. doi: 10.1111/mmi.12058 23062283 10.1111/mmi.12058

[22] Singh SK, Parveen S, SaiSree L, Reddy M. Regulated proteolysis of a cross-link–specific peptidoglycan hydrolase contributes to bacterial morphogenesis. Proc Natl Acad Sci USA. 2015;112: 10956–10961. doi: 10.1073/pnas.1507760112 26283368 10.1073/pnas.1507760112 PMC4568209

[23] Tomasz A. The mechanism of the irreversible antimicrobial effects of penicillins: how the beta-lactam antibiotics kill and lyse bacteria. Annu Rev Microbiol. 1979;33: 113–137. doi: 10.1146/annurev.mi.33.100179.000553 40528 10.1146/annurev.mi.33.100179.000553

[24] Höltje J-V. Growth of the Stress-Bearing and Shape-Maintaining Murein Sacculus of Escherichia coli. Microbiol Mol Biol Rev. 1998;62: 181–203. 9529891 10.1128/mmbr.62.1.181-203.1998 PMC98910

[25] Vollmer W, Joris B, Charlier P, Foster S. Bacterial peptidoglycan (murein) hydrolases. FEMS Microbiol Rev. 2008;32: 259–286. doi: 10.1111/j.1574-6976.2007.00099.x 18266855 10.1111/j.1574-6976.2007.00099.x

[26] van Opijnen T, Camilli A. Transposon insertion sequencing: a new tool for systems-level analysis of microorganisms. Nat Rev Microbiol. 2013;11: 435–442. doi: 10.1038/nrmicro3033 23712350 10.1038/nrmicro3033 PMC3842022

[27] Baba T, Ara T, Hasegawa M, Takai Y, Okumura Y, Baba M, et al Construction of Escherichia coli K-12 in-frame, single-gene knockout mutants: the Keio collection. Mol Syst Biol. 2006;2: 2006 0008. doi: 10.1038/msb4100050 16738554 10.1038/msb4100050 PMC1681482

[28] Bergman JM, Hammarlöf DL, Hughes D. Reducing ppGpp Level Rescues an Extreme Growth Defect Caused by Mutant EF-Tu. Jan E, editor. PLoS ONE. 2014;9: e90486 doi: 10.1371/journal.pone.0090486 24587376 10.1371/journal.pone.0090486 PMC3938759

[29] Majdalani N, Gottesman S. The Rcs phosphorelay: a complex signal transduction system. Annu Rev Microbiol. 2005;59: 379–405. doi: 10.1146/annurev.micro.59.050405.101230 16153174 10.1146/annurev.micro.59.050405.101230

[30] Potrykus K, Cashel M. (p)ppGpp: still magical? Annu Rev Microbiol. 2008;62: 35–51. doi: 10.1146/annurev.micro.62.081307.162903 18454629 10.1146/annurev.micro.62.081307.162903

[31] Cho S-H, Szewczyk J, Pesavento C, Zietek M, Banzhaf M, Roszczenko P, et al Detecting Envelope Stress by Monitoring β-Barrel Assembly. Cell. 2014;159: 1652–1664. doi: 10.1016/j.cell.2014.11.045 25525882 10.1016/j.cell.2014.11.045

[32] Konovalova A, Perlman DH, Cowles CE, Silhavy TJ. Transmembrane domain of surface-exposed outer membrane lipoprotein RcsF is threaded through the lumen of beta-barrel proteins. Proc Natl Acad Sci USA. 2014;111: E4350–E4358. doi: 10.1073/pnas.1417138111 25267629 10.1073/pnas.1417138111 PMC4205638

[33] Konovalova A, Mitchell AM, Silhavy TJ. A lipoprotein/β-barrel complex monitors lipopolysaccharide integrity transducing information across the outer membrane. elife. 2016;5: 5312. 10.7554/eLife.15276 PMC4942254 27282389

[34] Uehara T, Park JT. Growth of Escherichia coli: significance of peptidoglycan degradation during elongation and septation. J Bacteriol. 2008;190: 3914–3922. doi: 10.1128/JB.00207-08 18390656 10.1128/JB.00207-08 PMC2395050

[35] Nichols RJ, Sen S, Choo YJ, Beltrao P, Zietek M, Chaba R, et al Phenotypic Landscape of a Bacterial Cell. Cell. 2011;144: 143–156. doi: 10.1016/j.cell.2010.11.052 21185072 10.1016/j.cell.2010.11.052 PMC3060659

[36] Firczuk M, Bochtler M. Folds and activities of peptidoglycan amidases. FEMS Microbiol Rev. 2007;31: 676–691. doi: 10.1111/j.1574-6976.2007.00084.x 17888003 10.1111/j.1574-6976.2007.00084.x

[37] Markovski M, Bohrhunter JL, Lupoli TJ, Uehara T, Walker S, Kahne DE, et al Cofactor bypass variants reveal a conformational control mechanism governing cell wall polymerase activity. Proc Natl Acad Sci USA. 2016;113: 201524538–4793. doi: 10.1073/pnas.1524538113 27071112 10.1073/pnas.1524538113 PMC4855605

[38] García del Portillo F, de Pedro MA. Differential effect of mutational impairment of penicillin-binding proteins 1A and 1B on Escherichia coli strains harboring thermosensitive mutations in the cell division genes ftsA, ftsQ, ftsZ, and pbpB. J Bacteriol. 1990;172: 5863–5870. 2211517 10.1128/jb.172.10.5863-5870.1990 PMC526905

[39] Hashimoto M, Ooiwa S, Sekiguchi J. Synthetic lethality of the lytE cwlO genotype in Bacillus subtilis is caused by lack of D,L-endopeptidase activity at the lateral cell wall. J Bacteriol. 2012;194: 796–803. doi: 10.1128/JB.05569-11 22139507 10.1128/JB.05569-11 PMC3272963

[40] Bisicchia P, Noone D, Lioliou E, Howell A, Quigley S, Jensen T, et al The essential YycFG two-component system controls cell wall metabolism in Bacillus subtilis. Molecular Microbiology. 2007;65: 180–200. doi: 10.1111/j.1365-2958.2007.05782.x 17581128 10.1111/j.1365-2958.2007.05782.x

[41] Dörr T, Davis BM, Waldor MK. Endopeptidase-mediated beta lactam tolerance. PLoS Pathog. 2015;11: e1004850 doi: 10.1371/journal.ppat.1004850 25884840 10.1371/journal.ppat.1004850 PMC4401780

[42] Pazos M, Peters K, Vollmer W. Robust peptidoglycan growth by dynamic and variable multi-protein complexes. Curr Opin Microbiol. 2017;36: 55–61. doi: 10.1016/j.mib.2017.01.006 28214390 10.1016/j.mib.2017.01.006

[43] Vollmer W, Rechenberg von M, Höltje JV. Demonstration of molecular interactions between the murein polymerase PBP1B, the lytic transglycosylase MltA, and the scaffolding protein MipA of Escherichia coli. J Biol Chem. 1999;274: 6726–6734. 10037771 10.1074/jbc.274.10.6726

[44] Romeis T, Höltje JV. Specific interaction of penicillin-binding proteins 3 and 7/8 with soluble lytic transglycosylase in Escherichia coli. J Biol Chem. 1994;269: 21603–21607. 8063800

[45] Rechenberg von M, Ursinus A, Höltje JV. Affinity chromatography as a means to study multienzyme complexes involved in murein synthesis. Microb Drug Resist. 1996;2: 155–157. doi: 10.1089/mdr.1996.2.155 9158739 10.1089/mdr.1996.2.155

[46] Legaree BA, Clarke AJ. Interaction of penicillin-binding protein 2 with soluble lytic transglycosylase B1 in Pseudomonas aeruginosa. J Bacteriol. 2008;190: 6922–6926. doi: 10.1128/JB.00934-08 18708507 10.1128/JB.00934-08 PMC2566182

[47] Typas A, Banzhaf M, van den Berg van Saparoea B, Verheul J, Biboy J, Nichols RJ, et al Regulation of peptidoglycan synthesis by outer-membrane proteins. Cell. 2010;143: 1097–1109. doi: 10.1016/j.cell.2010.11.038 21183073 10.1016/j.cell.2010.11.038 PMC3060616

[48] Paradis-Bleau C, Markovski M, Uehara T, Lupoli TJ, Walker S, Kahne DE, et al Lipoprotein cofactors located in the outer membrane activate bacterial cell wall polymerases. Cell. 2010;143: 1110–1120. doi: 10.1016/j.cell.2010.11.037 21183074 10.1016/j.cell.2010.11.037 PMC3085243

[49] Egan AJF, Jean NL, Koumoutsi A, Bougault CM, Biboy J, Sassine J, et al Outer-membrane lipoprotein LpoB spans the periplasm to stimulate the peptidoglycan synthase PBP1B. Proc Natl Acad Sci USA. 2014;111: 8197–8202. doi: 10.1073/pnas.1400376111 24821816 10.1073/pnas.1400376111 PMC4050580

[50] Miller J. Experiments in Molecular Genetics. New York: Cold Spring Harbor Laboratory; 1972.

[51] Guyer MS, Reed RR, Steitz JA, Low KB. Identification of a sex-factor-affinity site in E. coli as gamma delta. Cold Spring Harb Symp Quant Biol. 1981;45 Pt 1: 135–140. 6271456 10.1101/sqb.1981.045.01.022

[52] Bernhardt TG, de Boer PAJ. Screening for synthetic lethal mutants in Escherichia coli and identification of EnvC (YibP) as a periplasmic septal ring factor with murein hydrolase activity. Molecular Microbiology. 2004;52: 1255–1269. doi: 10.1111/j.1365-2958.2004.04063.x 15165230 10.1111/j.1365-2958.2004.04063.x PMC4428336

