# Supplementary figures and images for: The mecillinam resistome reveals a role for peptidoglycan endopeptidases in stimulating cell wall synthesis in Escherichia coli
Source: PLoS Genet. 2017 Jul 27;13(7):e1006934. doi: 10.1371/journal.pgen.1006934 (PMC5549755; doi:10.1371/journal.pgen.1006934)

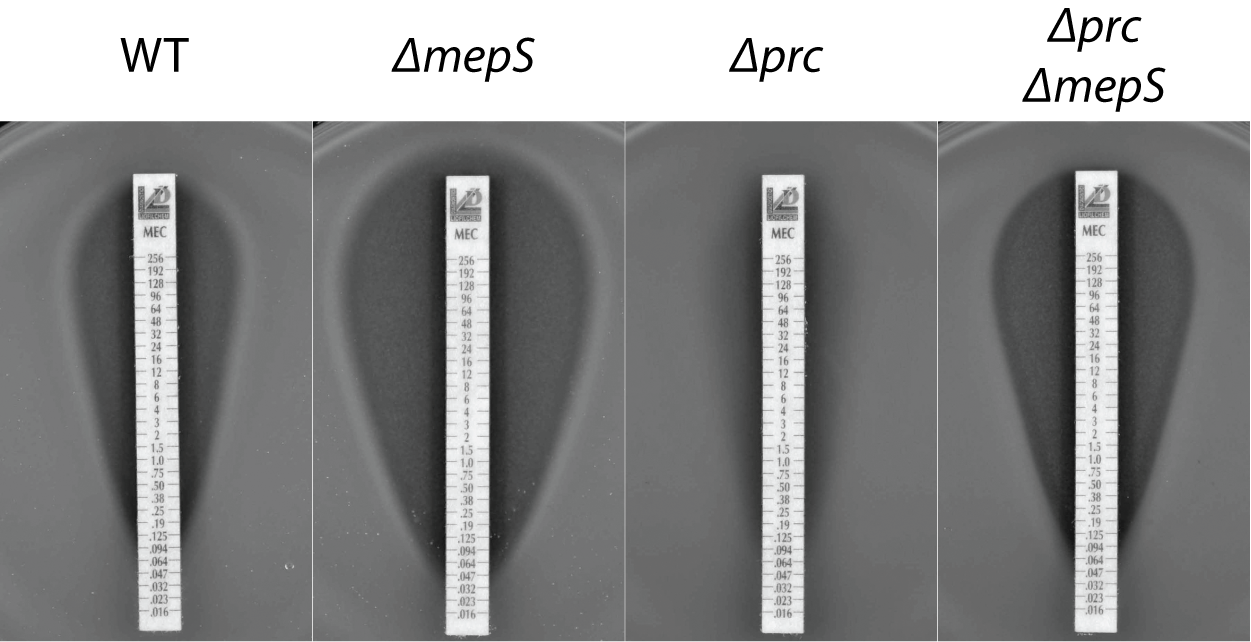

Supplement: S1 Fig — Lawns of TB28/pTB63 [WT/ftsZup] and its indicated derivatives were plated in soft agar and incubated with mecillinam test strips as in Fig 3. Note that loss of Prc function results in resistance and that sensitivity is restored by MepS inactivation. (TIF) [file pgen.1006934.s001.tif]

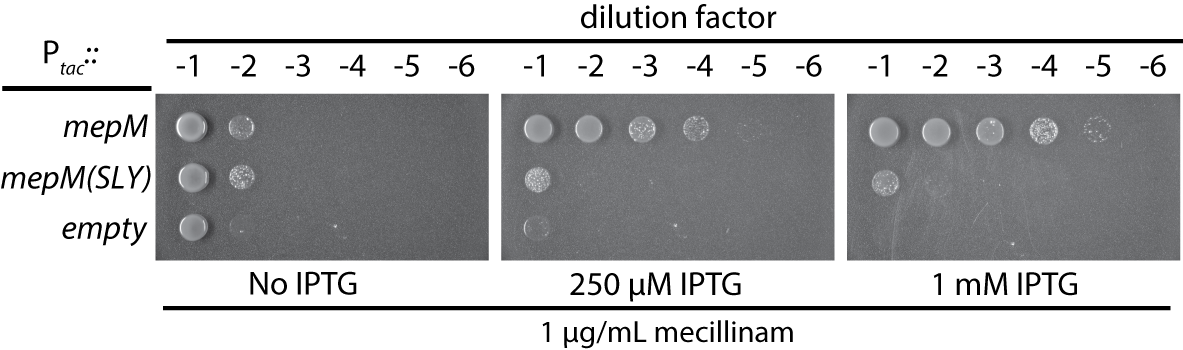

Supplement: S2 Fig — Overnight cultures of MG1655/pTB63 with plasmids pTK1 [Ptac::mepM], pTKD8 [Ptac::mepM(SLY)], or pHC800 [Ptac::empty] were serially diluted and spotted on LB agar containing mecillinam and IPTG at the indicated concentrations. The plates were incubated at 30°C for 24 hrs and photographed. The mepM(SLY) gene encodes MepM with residues HLH(393–395) converted to SLY. This substitution mimics the defective LytM active site of the related EnvC protein. (TIF) [file pgen.1006934.s002.tif]
