# Supplementary material for: The mecillinam resistome reveals a role for peptidoglycan endopeptidases in stimulating cell wall synthesis in Escherichia coli
Source: PLoS Genet. 2017 Jul 27;13(7):e1006934. doi: 10.1371/journal.pgen.1006934 (PMC5549755; doi:10.1371/journal.pgen.1006934)
Supplement: S1 Text — (DOCX) [file pgen.1006934.s003.docx]

**Plasmid constructions**

Plasmid construction procedures are given below. Restriction sites encoded in primers used are italicized and underlined in the primer sequences given in the description.

**pTK1, pTK2, pTK4, and pTKD4**

The *mepM* (pTK1), *mepS* (pTK2), *mepA* (pTK4), and *pbpG* (pTKD4) genes were amplified with their native ribosome-binding sequences using primer pairs 5’-GCTA*TCTAGA*TATGCGAGCTGCCTGAAAGGAG-3’ / 5’-GCTA*GGATCC*TTAATCAAACCGTAGCTGCGGC-3’ (*mepM*), 5’-GCTA*TCTAGA*GATATTTGTCGTTAAGGACTTC-3’ / 5’-GCTA*GGATCC*TTAGCTGCGGCTGAGAACCCGG-3’ (*mepS*), 5’-GCTA*TCTAGA*GCCGATGTGAAGACTGATATTC-3’ / 5’-GCTA*AAGCTT*TTAGATCACGTGCTCATCCAG-3’ (*mepA*), and 5’-GCTA*TCTAGA*GAACCACTATCTGAATGCTC-3’ / 5’-GCTA*AAGCTT*AATCGTTCTGTGCCGTCTG-3’ (*pbpG*).

The resulting PCR products were digested with XbaI/BamHI (*mepM* and *mepS*) or XbaI/HindIII (*mepA* and *pbpG*) and ligated to pHC800 digested with the same enzymes.

**pTKD8**

To clone a catalytically inactive *mepM* allele, HLH(393-395) residues that are important for the retention of Zn and thus required for MepM catalytic activity were mutated to SLY. Amino acid change was designed based on the corresponding sequence of a degenerate LytM family protein EnvC. *mepM(SLY)* allele was amplified by overlap extension by using the mepM primers decribed above and mutagenic primers 5’-AACCGGGCCG**AGC**CTG**TAT**TATGAAGTATGGATAAACCAGCAGG-3’ and 5’-ATACTTCATA**ATA**CAG**GCT**CGGCCCGGTTGAACGTCCGGTATTA-3’. 3’ (mutated bases denoted in bold). The resulting PCR product was cloned in XbaI/BamHI-digested pHC800.

**pGL65-68**

For construction of pGL65-68, coding sequences of truncated *relA*, *mepS*, *mepS(C68A)*, and *rcsF* were cloned with the ribosome binding sequence of the T7 phi10 gene (AAGGAG, underlined) to attain strong expression from chromosomally-integrated plasmids.

**pGL65:**

The site of a *relA* truncation resulting in mecillinam resistance, and presumably the overproduction of ppGpp, was determined from transposon sequencing results. In the presence of mecillinam, transposon insertions were enriched in *relA* at base 1096 by >1000 fold. The corresponding *relA*::EZTn5 insertion mutant was recreated using lambda recombineering. The EZ-Tn5<KAN-2> sequence was first amplified using a primer pair 5’-ACTGGAAATATAAAGAGGGCGCGGCTGCTGGCGGCGCTGTCTCTTATACACATCTCAACCATCATCGATGAATTG-3’ and 5’-GCAGCCAGGCAATCCGGTCTTCATGTCCCGAACGTGCGCCGCCAGCTGTCTCTTATACACATCTCAACCCTGAAGCTTGCATGC-3’ , electroporated into TB10, and recombinants selected on LB Kan. Underlined portions of the primer sequence correspond to *relA* gene homology for recombineering. The resulting *relA*::Tn5 mutant was then sequenced and its ability to confer mecillinam resistance was confirmed.

Next, the *relA* truncation (i.e. *relA* bases (1-1096) + Tn5 sequence up until a stop codon was encountered, was amplified using a primer pair 5’-CTAGAAATAATTTTGTTTAACTTTAAGAAGGAGATATACATATGGTTGCGGTAAGAAGTG-3’ and 5’-ATATTATCGTGAGATCGATAAACACCCCTTGTATTACTGT-3. The resulting PCR product was cloned in XbaI/HindIII-digested pHC432 via isothermal assembly to generate pGL65.

**pGL66-67:**

*mepS* was amplified using a primer pair 5’- GAGCGGATAACAATTCCCCTTCTAGAAATAATTTTGTTTAACTTTAAGAAGGAGATATACATATGGTCAAATCTCAACCGA -3’ and 5’- ATATTATCGTGAGATCGATAGGATCCTTAGCTGCGGCTGAGAACCCGG-3’. *mepS(C68A)* was amplified by overlap extension PCR using the *mepS* primers and mutagenic primers 5’-AAAAAAGGTATCGAT**GCG**TCTGGTTTCGTACAGCGTACATTCC-3’ and 5’-CTGTACGAAACCAGA**CGC**ATCGATACCTTTTTTAGTGCTGCCG-3’ (mutated bases denoted in bold). The resulting PCR products were cloned in XbaI/HindIII-digested pHC739 via isothermal assembly to generate pGL66 and pGL67.

**pGL68:**

*rcsF* was amplfied using a primer pair 5’-AAAATCTAGAAATAATTTTGTTTAACTTTAAGAAGGAGATATACATATGCGTGCTTTACCGATC-3’ and 5’-AAAAAAGCTTTCATTTCGCCGTAATGTT-3’, digested via XbaI/HindIII and ligated to pHC739 digested with XbaI/HindIII.
