# Supplementary material for: The mecillinam resistome reveals a role for peptidoglycan endopeptidases in stimulating cell wall synthesis in Escherichia coli
Source: PLoS Genet. 2017 Jul 27;13(7):e1006934. doi: 10.1371/journal.pgen.1006934 (PMC5549755; doi:10.1371/journal.pgen.1006934)
Supplement: S3 Table — (PDF) [file pgen.1006934.s006.pdf]

Table S3. Strains used in this study.

Strain	Genotype[a]	Source/Reference[b]
DH5α	*F– hsdR17 deoR recA1 endA1 phoA supE44 thi-1 gyrA96 relA1 Δ(lacZYA-argF)U169 ϕ80dlacZΔM15*	Gibco BRL
TB10	MG1655 *λΔcro-bio nad::Tn10*	[1]
MG1655	*rph-1 ilvG rfb-50*	[2]
TB28	*MG1655 ΔlacIZYA::frt*	[3]
GL38	MG1655 Δ*rcsB::frt*	This study[a]
GL44	MG1655 Δ*relA::frt*	This study[a]
GL74	MG1655 Δ*rfaP::Kan*^{R}	This study[a]
GL76	MG1655 Δ*rfaH:: Kan*^{R}	This study[a]
GL82	MG1655 Δ*prc:: Kan*^{R}	This study[a]
GL112	MG1655 Δ*rnt:: Kan*^{R}	This study[a]
GL120	MG1655 Δ*efp::Kan*^{R}	This study[a]
GL122	MG1655 Δ*sspA::Kan*^{R}	This study[a]
AG04	MG1655 Δ*lpp::Kan*^{R}	This study[a]
HC408	MG1655 Δ*slt::Kan*^{R}	This study[a]
GL101	MG1655 Δ*arcA::Kan*^{R}	This study[a]
GL90	MG1655 Δ*gcvR::Kan*^{R}	This study[a]
GL92	MG1655 Δ*crr::Kan*^{R}	This study[a]
GL70	MG1655 Δ*lepA::Kan*^{R}	This study[a]
GL72	MG1655 Δ*trxB::Kan*^{R}	This study[a]
GL124	MG1655 Δ*cysE::Kan*^{R}	This study[a]
GL78	MG1655 Δ*pgm::Kan*^{R}	This study[a]
GL84	MG1655 Δ*nlpI::Kan*^{R}	This study[a]
GL80	MG1655 Δ*opgH::Kan*^{R}	This study[a]
GL206	MG1655 Δ*nlpD::Kan*^{R}	This study[a]

GL66	MG1655 Δ*tufA::Kan*^{R}	This study[a]
GL204	MG1655 Δ*rcsB::frt* Δ*rfaP::Kan*^{R}	This study[a]
GL205	MG1655 Δ*rcsB::frt* Δ*nlpD::Kan*^{R}	This study[a]
GL203	MG1655 Δ*rcsB::frt* Δ*lpp::Kan*^{R}	This study[a]
GL207	MG1655 Δ*relA::frt* Δ*tufA::Kan*^{R}	This study[a]
GL243	MG1655 Δ*relA::frt* Δ*efp::Kan*^{R}	This study[a]
GL62	MG1655 Δ*rcsB::frt* Δ*slt::Kan*^{R}	This study[a]
GL180	MG1655 Δ*relA::frt* Δ*slt::Kan*^{R}	This study[a]
GL68	MG1655 Δ*mepS::frt*	This study[a]
GL67	MG1655 Δ*ponB::frt*	This study[a]
TB153	TB28 *ΔmepS::Kan*^{R}	This study[a]
DL84	TB28 *Δprc::frt*	This study[a]
HC604	TB28 *Δprc::frt ΔmepS::Kan*^{R}	This study[a]
TU278	TB28 Δ*lysA::frt* Δ*ampD::frt*	[4]
HC533	MG1655 Δ*lysA::frt* Δ*ampD::frt* Δ*ponA::frt* Δ*pbpC::frt* Δ*mtgA::frt* ^{MS}*ponB*	[5]

[a] The indicated strains were generated by P1 transduction by moving the indicated gene replacement alleles from the Keio collection [26] to the desired genetic background. In cases were the allele was unavailable or incorrect in our copy of the collection, equivalent mutants were regenerated by recombineering using strain TB10 and then transduced from this background. Kan^R cassettes are flanked by *frt* sites for removal by FLP recombinase. An *frt* scar remains following removal of the cassette using FLP expressed from pCP20.

[b] 1. Johnson JE, Lackner LL, Hale CA, de Boer PAJ. ZipA is required for targeting of DMinC/DicB, but not DMinC/MinD, complexes to septal ring assemblies in Escherichia coli. J Bacteriol. 2004;186: 2418–2429.
2. Guyer MS, Reed RR, Steitz JA, Low KB. Identification of a sex-factor-affinity site in E. coli as gamma delta. Cold Spring Harb Symp Quant Biol. 1981;45 Pt 1: 135–140.
3. Bernhardt TG, de Boer PAJ. Screening for synthetic lethal mutants in Escherichia coli and identification of EnvC (YibP) as a periplasmic septal ring factor with murein hydrolase activity. Molecular Microbiology. 2004;52: 1255–1269. doi:10.1111/j.1365-2958.2004.04063.x
4. Cho H, Uehara T, Bernhardt TG. Beta-lactam antibiotics induce a lethal malfunctioning of the bacterial cell wall synthesis machinery. Cell. 2014;159: 1300–1311. doi:10.1016/j.cell.2014.11.017
5. Cho H, Wivagg CN, Kapoor M, Barry Z, Rohs PDA, Suh H, et al. Bacterial cell wall biogenesis is mediated by SEDS and PBP polymerase families functioning semi-autonomously. Nat Microbiol. 2016;1: 16172. doi:10.1038/nmicrobiol.2016.172
