# Supplementary material for: The mecillinam resistome reveals a role for peptidoglycan endopeptidases in stimulating cell wall synthesis in Escherichia coli
Source: PLoS Genet. 2017 Jul 27;13(7):e1006934. doi: 10.1371/journal.pgen.1006934 (PMC5549755; doi:10.1371/journal.pgen.1006934)
Supplement: S4 Table — (PDF) [file pgen.1006934.s007.pdf]

Table S4. Plasmids used in this study.

Plasmid	Genotype[a]	Origin	Source/Reference[b]
pCP20	*bla cat* cI875 *repA*(Ts) P_{R}::*flp*	pSC101	[1]
pInt-ts	*bla* cI875 *repA*(Ts) P_{R}::int^{λ}	pSC101	[2]
pTB63	*tetA* P_{native}::*ftsQAZ*	pSC101	[3]
pHC432	*cat attλ* P_{ara}::*slmA*	R6K	[4]
pHC739	*cat attλ* P_{tac}::*sulA*	R6K	[5]
pHC800	*cat* P_{tac}::*empty*	ColE1	[5]
pTK1	*cat* P_{tac}::*native RBS-mepM*	ColE1	This study
pTK2	*cat* P_{tac}::*native RBS-mepS*	ColE1	This study
pTK4	*cat* P_{tac}::*native RBS-mepA*	ColE1	This study
pTKD4	*cat* P_{tac}::*native RBS-pbpG*	ColE1	This study
pTKD8	*cat* P_{tac}::*native RBS-mepM(HLH[393-395]>SLY)*	ColE1	This study
pHC859	*tetAR attHK* P_{tac}::*sulA*	R6K	[6]
pGL65	*cat attλ* P_{ara}::*phi10 RBS-relA(1-365)*	R6K	This study
pGL66	*cat attλ* P_{tac}::*phi10 RBS-mepS*	R6K	This study
pGL67	*cat attλ* P_{tac}::*phi10 RBS-mepS(C68A)*	R6K	This study
pGL68	*cat attλ* P_{tac}::*phi10 RBS-rcsF*	R6K	This study
pGL69	*cat attλ* P_{ara}::*empty*	R6K	This study
pGL70	*cat attλ* P_{tac}::*empty*	R6K	This study

[a] P_{lac}, P_{T7}, P_{R}, P_{ara}, and P_{tac} indicate the lactose, phage T7, λR, arabinose promoters, and the synthetic fusion promoter of tryptophan and lacUV5, respectively. Where relevant, the status of the ribosome binding site (RBS), either native or the phi10 RBS from phage T7, is indicated.

[b] 1. Datsenko KA, Wanner BL. One-step inactivation of chromosomal genes in Escherichia coli K-12 using PCR products. Proc Natl Acad Sci USA. 2000;97: 6640–6645. doi:10.1073/pnas.120163297

2. Haldimann A, Wanner BL. Conditional-replication, integration, excision, and retrieval plasmid-host systems for gene structure-function studies of bacteria. J Bacteriol. 2001;183: 6384–6393. doi:10.1128/JB.183.21.6384-6393.2001

3. Bendezú FO, de Boer PAJ. Conditional lethality, division defects, membrane involution, and endocytosis in mre and mrd shape mutants of Escherichia coli. J Bacteriol. 2008;190: 1792–1811. doi:10.1128/JB.01322-07

4. Cho H, Bernhardt TG. Identification of the SlmA active site responsible for blocking bacterial cytokinetic ring assembly over the chromosome. PLoS Genet. 2013;9: e1003304. doi:10.1371/journal.pgen.1003304
5. Cho H, Uehara T, Bernhardt TG. Beta-lactam antibiotics induce a lethal malfunctioning of the bacterial cell wall synthesis machinery. Cell. 2014;159: 1300–1311. doi: 10.1016/j.cell.2014.11.017
6. Yunck R, Cho H, Bernhardt TG. Identification of MltG as a potential terminase for peptidoglycan polymerization in bacteria. Molecular Microbiology. 2015;99: 700–718. doi:10.1111/mmi.13258
